# Alterations of endocannabinoid signaling and microglia reactivity in the retinas of AD‐like mice precede the onset of hippocampal β‐amyloid plaques

**DOI:** 10.1111/jnc.16256

**Published:** 2024-11-18

**Authors:** Annamaria Tisi, Lucia Scipioni, Giulia Carozza, Lucia Di Re, Giacomo Cimino, Camilla Di Meo, Sakthimala Palaniappan, Francesco Della Valle, Federico Fanti, Giacomo Giacovazzo, Dario Compagnone, Rita Maccarone, Sergio Oddi, Mauro Maccarrone

**Affiliations:** ^1^ Department of Biotechnological and Applied Clinical Sciences University of L'Aquila L'Aquila Italy; ^2^ Laboratory of Lipid Neurochemistry, European Center for Brain Research (CERC) Santa Lucia Foundation IRCCS Rome Italy; ^3^ Department of Veterinary Medicine University of Teramo Teramo Italy; ^4^ Department of Bioscience and Technology for Food, Agriculture and Environment University of Teramo Teramo Italy

**Keywords:** Alzheimer's disease, bioactive lipids, endocannabinoids, neuroinflammation, retina

## Abstract

Extra‐cerebral manifestations of Alzheimer's disease (AD) develop in the retina, which is, therefore, considered a “window to the brain”. Recent studies demonstrated the dysregulation of the endocannabinoid (eCB) system (ECS) in AD brain. Here, we explored the possible alterations of ECS and the onset of gliosis in the retina of AD‐like mice. Tg2576 (TG) mice overexpressing the amyloid precursor protein (APP) were used at the age of 12 months, when hippocampal β‐amyloid plaques had not been developed yet. Analysis of retinal gliosis showed a significant increase in the number of IBA1 (+) microglia cells in TG versus wild type (WT). Gliosis was not associated with retinal β‐amyloid plaques, evident retinal degenerative signatures, or excitotoxicity; instead, oxidative stress burden was observed as increased acrolein levels. Analysis of the ECS (receptors/metabolic enzymes) through western blotting (WB) revealed the up‐regulation of cannabinoid receptor 2 (CB_2_) and monoacylglycerol lipase (MAGL), the enzyme responsible for the degradation of 2‐arachidonoylglycerol (2‐AG), in TG retinas. Fluorescence intensity analysis of anti‐CB_2_ and anti‐MAGL immuno‐stained cryosections was consistent with WB, showing their up‐regulation throughout the retinal layers. No statistically significant differences were found for the other enzymes/receptors of the ECS under study. However, linear regression analysis for individual animals showed a significant correlation between CB_2_ and fatty acid amide hydrolase (FAAH), diacylglycerol lipase α/β (DAGLα/β), and APP; instead, a significant negative correlation was found between MAGL and APP. Finally, ultra‐performance liquid chromatography–tandem mass spectrometry (UPLC–MS/MS) demonstrated a significant reduction of 2‐AG in TG retinas (~0.34 ng/mg) compared to WT (~1.70 ng/mg), while a trend toward increase was found for the other eCB anandamide (AEA). Overall, our data indicate that gliosis and ECS dysregulation—in particular of CB_2_, MAGL and 2‐AG—occur in the retina of AD‐like mice before retinal degeneration and development of hippocampal β‐amyloid plaques.

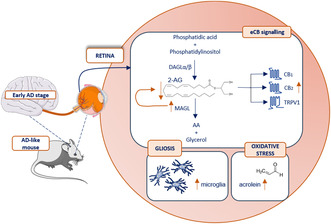

## Graphical abstract

Extra‐cerebral manifestations of Alzheimer's disease (AD) have been observed in the eye. Here, we analyzed the retina of AD‐like mice (Tg2576 model) at an early stage of the brain pathology. We discovered important alterations of the endocannabinoid (eCB) system, particularly of 2‐AG metabolism/signaling, alongside microglia reactivity and oxidative stress burden in the retina of those mice. These events occurred well before the development of hippocampal β‐amyloid plaques, indicating that such retinal processes are early events in the pathogenesis of AD in Tg2576 mice.

**Abbreviations**
2‐AG: 2‐arachidonoylglycerol
AD: Alzheimer's disease
AEA: anandamide, N‐arachidonoylethanolamine
APP: amyloid precursor protein
BSA: bovine serum albumin
CB_1_
: cannabinoid receptor 1
CB_2_
: cannabinoid receptor 2
CNS: central nervous system
cSLO: confocal scanning laser ophthalmoscopy
DAGLα/β: diacylglycerol lipase α/β
eCB: endocannabinoid
ECS: endocannabinoid system
FAAH: fatty acid amide hydrolase
GAPDH: glyceraldehyde‐3‐phosphate dehydrogenase
GCL: ganglion cell layer
GFAP: glial fibrillary acidic protein
HRP: horse radish peroxidase
IBA‐1: ionized calcium‐binding adapter molecule 1
IF: immunofluorescence
IL‐1: interleukin‐1
INL: inner nuclear layer
IPL: inner plexiform layer
MAGL: monoacylglycerol lipase
NAPE‐PLD: N‐acylphosphatidylethanolamines‐specific phospholipase D
O.N.: optic nerve
OCT: optical coherence tomography
ONL: outer nuclear layer
OPL: outer plexiform layer
OS: outer segments
PFA: paraformaldehyde
RT: room temperature
TG: transgenic
TRPV1: transient receptor potential vanilloid type 1
UPLC‐MS/MS: ultra‐performance liquid chromatography‐mass spectrometry
WB: western blotting
WT: wild type

## 1 INTRODUCTION

Alzheimer's disease (AD) is a degenerative condition that progressively impairs basic cognitive functions, especially memory and thinking abilities, with death as an inevitable outcome within 5–12 years from the onset of symptoms (2021 Alzheimer's disease facts and figures, 2021). Although it is generally considered that Aβ aggregation is a primary event in initiating AD pathogenesis, recent studies suggest that such a cause alone is not sufficient. Indeed, synaptic degeneration and progression of AD pathology are further affected by the activation of glial cells, microglia and astrocytes, which have now been recognized as key players in AD pathogenesis (Tzioras et al., 2023). Intriguingly, histology investigations demonstrated that also the retinas of AD patients display inflammatory processes as does the brain (Grimaldi et al., 2019). Specifically, astrocyte and microglial activity were observed in *post‐mortem* retinal tissues of AD patients as compared to control participants, associated with Aβ plaques, tau tangles, and neurodegeneration. The retinal tissues of AD patients also exhibit elevated amounts of osteopontin, complement component 3, and interleukin‐1 (IL‐1; Grimaldi et al., 2019). In addition, many patients show visual impairment such as visual field defects, changes in contrast sensitivity, and reduced color vision discrimination. Interestingly, visual defects seem to manifest before the first cognitive signs of AD (Jorge et al., 2020). On this basis, the retina, often regarded as a “window to the brain”, offers a unique opportunity to gain insights into the intricacies of neurological function. With its neural extension, shared developmental origins, and analogous composition to the brain, the retina serves as a direct link between the visual system and the central nervous system (CNS; Donato et al., 2023; London et al., 2013). Moreover, given the accessibility and transparency of the cornea, a variety of technologies enable non‐invasive, high‐resolution imaging of the retina such as optical coherence tomography (OCT) and confocal scanning laser ophthalmoscopy (cSLO; London et al., 2013; Webb et al., 1987).

In the context of the neuroinflammatory processes of AD, the endocannabinoid system (ECS) is emerging as a key player. The ECS is a lipid signaling widely expressed throughout the body with manifold physiological functions (Maccarrone, 2020; Oddi et al., 2023). The major endocannabinoids (eCBs) include the 2‐arachidonoylglycerol (2‐AG; an ester of AA) and the *N*‐arachidonoylethanolamine or anandamide (AEA; an amide of AA); in addition to eCBs, the ECS comprises their biosynthetic and degradative enzymes, and their receptors. The main enzymes responsible for the biosynthesis of 2‐AG and AEA are diacylglycerol lipases (DAGL α and β) and *N*‐acylphosphatidylethanolamine‐hydrolyzing phospholipase D (NAPE‐PLD) respectively, while the main enzymes responsible for their degradation are monoacylglycerol lipase (MAGL) and fatty acid amide hydrolase (FAAH). The biological activity of the eCBs may be exerted through the binding to their receptors, which include the cannabinoid receptor 1 (CB_1_) and 2 (CB_2_) and the transient receptor potential vanilloid type 1 (TRPV1; Maccarrone et al., 2023). The eCBs have recently been recognized as “immunomodulatory mediators”, and alterations in their levels, as well as in other ECS components, are involved in human diseases—neurodegenerative disorders included—characterized by chronic inflammation (Leuti et al., 2020). For instance, altered CB_1_ localization was found in the brain of AD‐like mice (Leuti et al., 2020). Also, CB_2_ was found to be implicated in AD pathogenesis, with CB_2_ levels significantly up‐regulated in AD brains, probably because of increased microglia number (Benito et al., 2003). Additionally, it has been found that the structure and/or function of the metabolic enzymes of eCBs are affected in AD brains. Accordingly, alterations in the levels of the main eCBs, AEA and 2‐AG, have been documented in the brain of AD‐like mice and *post‐mortem* tissues from AD patients (Mulder et al., 2011; Vázquez et al., 2015). Unsurprisingly, the ECS has emerged as an important therapeutic target to treat neurodegenerative disorders like AD.

Against this background, it is apparent that alterations in the ECS are closely linked to AD pathogenesis and its neuroinflammatory processes in the brain. However, the actual role of ECS in AD remains to be fully elucidated, and its possible involvement in AD‐associated retinal inflammation has never been investigated. Here, we sought to fill this knowledge gap and advance our understanding of the complex pathological underpinnings of AD.

## 2 MATERIALS AND METHODS

### 2.1 Animals

All experiments were conducted in accordance with the Italian and European laws on the use of animals for research (DLGs n.26 of 04/03/2014, European Communities Council Directive 2010/63/UE), and were authorized by the Italian Ministry of Health (authorization n. 421/2019‐PR). Specifically, in this study we used heterozygous transgenic Tg2576 (TG) mice, overexpressing human mutant (K670N/M671L) amyloid precursor protein (APP) transgene (hAPPK670N/M671L) under transcriptional control of the hamster prion promoter. Heterozygous Tg2576 mice were purchased from Taconic Biosciences (strain: B6;SJL‐Tg(APPSWE)2576Kha; RRID:IMSR_TAC:1349) and were crossed with C57BL/6J (RRID:IMSR_CRL:027) × SJL (RRID:IMSR_CRL:478) hybrid female mice. All animals were genotyped as previously described (Scipioni et al., 2023). The housing of the animals was at controlled temperature (19–22°C) and humidity (55%) with dim cyclic light settings (12‐h:12‐h light: dark). Mice were kept in cages containing up to 5 littermates, while male TG were kept in isolated cages because of the aggressive phenotype, as recommended. Water and food were available ad libitum. The purpose of the study was to investigate the retinas of Tg2576 at an early stage of the AD‐like phenotype. Therefore, the analysis was performed on male and female mice at the age of 12 months with an average weight of 40 g. This time point was selected because Tg2576 mice exhibit a slow progression of the pathology and 12‐month‐old Tg2576 are generally considered at an early stage of the disease (Puzzo et al., 2015). Moreover, since AD is considered an age‐related neurodegenerative disease, the use of aged mice—which still do not exhibit late‐stage‐related AD signatures—allowed us to also include age‐related processes, thus mimicking in a more realistic way the patients' conditions. *N* = 31 mice aged 12 months were used (WT: *n* = 14; TG: *n* = 17). A small group of 3 and 6 months old WT and TG mice (*n* = 12: 3/genotype/time point) was also included for screening of the temporal pattern of eCB levels. Overall, a total number of 43 animals were included in the study. No animals were excluded.

### 2.2 Extraction of retinal proteins

For tissue collection, the mice were decapitated following intraperitoneal (IP) anesthesia using a solution of ketamine and xylazine at a dosage of 100/10 mg/kg ketamine: (Nimatek im sc ev 10 mL 100 mg/mL, cat. no. 104661020), xylazine (Rompun iniet fl soluz 2%, cat. no. 100390018), and 14.25% ethanol (Sigma‐Aldrich, cat. no.1.08543) in 0.9% NaCl (PanReac AppliChem ITW Reagents, cat. no. A2942). Retinal samples (2 retinas per sample) were dissected and pooled to have enough protein quantity for western blotting. They were homogenized in 50 μL of lysis buffer (50 mM Tris–HCl (Sigma‐Aldrich, cat. no. T1503 pH 7.5), 1% Triton X‐100, (Sigma‐Aldrich, cat. no. X100), 0.1% SDS (Bio‐Rad, cat. no. 1610302), EDTA 5 Mm (Millipore, cat. no. 4005), Halt Protease and Phosphatase Inhibitor Cocktail (ThermoFisher Scientific, cat. no. 78440). The suspension was incubated for 20 min in ice, followed by centrifugation at 17 968 *g* for 20 min at 4°C. The supernatant was transferred to a new clean centrifuge tube and stored at −80°C. The Bradford colorimetric method (Bio‐Rad, cat. no. 5000205) was used to determine the total protein concentration.

### 2.3 Western blot

Protein expression of ECS components was analyzed by western blotting (WB).

Forty micrograms of the protein extracts were processed at 200 V for 20 min on a Bolt 4%–12% Bis‐Tris Plus (ThermoFisher Scientific, cat. no. NW04120BOX). The proteins were transferred to the PVDF membrane (Millipore, cat. no. IB24001) using the iBlot 2 Dry Blotting System (Invitrogen, cat. no. IB21001). The membrane was rapidly washed for 5 min in TBS‐T (10 mM Tris–HCl, (Sigma‐Aldrich, cat. no. T1503) 150 mM NaCl (PanReac AppliChem ITW Reagents, cat. no. A2942), 0.1% Tween 20 (Sigma‐Aldrich, cat. no. P9416), pH 7.6) and aspecific bindings were blocked with 5% nonfat dry milk (PanReac AppliChem ITW Reagents, cat. no. A0830) in TBS‐T for 1 h at room temperature (RT) on a shaker platform. Subsequently, the membranes were incubated with respective primary antibodies (CB_1_, CB_2_, TRPV1, DAGLα, DAGLβ, MAGL, NAPE‐PLD, and FAAH) overnight at 4°C diluted in 5% nonfat dry milk (PanReac AppliChem ITW Reagents, cat. no. A0830) in TBS‐T (a list of all the antibodies utilized in this study is summarized in Table 1). Then, the membranes were washed 3 times for 5 min with TBS‐T and incubated for 1 h at RT with the specific horse radish peroxidase (HRP) conjugated secondary antibody (anti‐rabbit or anti‐mouse) diluted 1:2000 in 5% fat‐free dry milk in TBS‐T. The stripping procedure was used when necessary.

**TABLE 1:** Antibodies used in the study.

Antibody	Use (WB/IF)	Company and cat. #.	Species	Dilution
CB_1_	WB	Abcam #ab259323	Rabbit	1:1000
CB_2_	WB	Cayman #101550	Rabbit	1:200
	IF			1:50
TRPV1	WB	OriGene #TA336871	Rabbit	1:1000
DAGLα	WB	Invitrogen #PA5‐23765	Rabbit	1:1000
DAGLβ	WB	Invitrogen #PA5‐26331	Rabbit	1:1000
MAGL	WB	Abcam #Ab24701	Rabbit	1:200
	IF			1:200
NAPE‐PLD	WB	Invitrogen #PA5‐115616	Rabbit	1:1000
FAAH	WB	Abcam #Ab54615	Mouse	1:1000
GAPDH	WB	Invitrogen #MA1‐16757	Mouse	1:1500
HRP Anti‐Mouse IgG	WB	Cayman #10004302	Goat	1:2000
HRP‐Anti‐Mouse IgG	IHC	Invitrogen #31430	Goat	1:300
HRP Anti‐Rabbit IgG	WB	Cayman #10004301	Goat	1:2000
IBA‐1	IF	Wako #019‐19 741	Rabbit	1:200
GFAP	IF	Cell Signaling #3670	Mouse	1:200
APP	WB	MerkMillipore #Mab348	Mouse	1:1000
β‐amyloid	IHC	Cell Signaling #15126	Mouse	1:200
Acrolein	IF	Abcam #Ab37110	Rabbit	1:250
ALEXA FLUOR 594	IF	Invitrogen #A11012	Goat anti‐rabbit	1:300
ALEXA FLUOR 488	IF	Invitrogen #A11008	Goat anti‐rabbit	1:300

*Note*: For abbreviations see the abbreviation list.

Finally, the membrane was washed as above and incubated in SuperSignal West Pico Pluschemiluminescent substrate for 30 s (ThermoFisher Scientific, cat. no. 34580). The bands were detected using a ChemiDoc XRSplus imaging system (Bio‐Rad Laboratories; RRID:SCR_019690). The optical densities of blot bands were analyzed and quantified by ImageJ (U.S. National Institutes of Health, Bethesda, MD, USA) software (RRID:SCR_003070), and were normalized versus glyceraldehyde‐3‐phosphate dehydrogenase (GAPDH) as the housekeeping protein. The results were expressed as the protein/GAPDH ratio and normalized to the control group.

### 2.4 Brain cryosections

Animals underwent deep anesthesia and were then perfused with a phosphate‐buffered solution (PBS; Sigma‐Aldrich, cat. no. P4417). The mice underwent intraperitoneal anesthesia using a mixture of ketamine and xylazine at a dosage of 100/10 mg/kg. The anesthesia comprised 25 mg/mL ketamine (cat. no. BP736), 2.5 mg/mL xylazine (cat. no. PHR3263), and 14.25% ethanol (Sigma‐Aldrich, cat. no.1.08543) in 0.9% NaCl (PanReac AppliChem ITW Reagents, cat. no. A2942), administered at a dosage of 0.30 mL/30 g mouse. Transcardiac perfusion was performed with a peristaltic pump for 10 min. Afterward, the brains were collected and fixed in 4% paraformaldehyde (PFA; Sigma‐Aldrich, cat. no.100496) for 48 h, then cryoprotected by immersion in 30% saccharose (Sigma‐Aldrich, cat. no. 0389) and frozen at −80°C. Coronal cryosections of 20 μm were then performed through a Leica CM1850 cryostat (GmbH, Nussloch, Germany; RRID:SCR_025401).

### 2.5 Retinal cryosections

The eyes enucleated for morphological analyses were fixed in 4% PFA (Sigma‐Aldrich, cat. no. 100496) for 6 h and then washed in 0.1 M PBS (Sigma‐Aldrich, cat. no. P4417; pH 7.4). The eyes were cryoprotected by immersion in 30% sucrose (Sigma‐Aldrich, cat. no. S7903) overnight, embedded in the Tissue Tek OCT (VWR, cat. no. 361603E) compound, and frozen in liquid nitrogen. Cryosections of 10 μm of thickness were made through a Leica CM1850 cryostat (GmbH, Nussloch, Germany; RRID:SCR_025401) and collected on gelatinand poly‐l‐lysine‐coated slides (Epredia, cat. no. J1800AMNZ). For immunofluorescence analysis, the sections crossing the optic nerve were selected.

### 2.6 Anti‐β‐amyloid immunohistochemistry (IHC)

For anti‐β‐amyloid immunohistochemistry (IHC), brain and retinal cryosections were incubated with 3% H_2_O_2_ for 1 h and then blocked with 5% bovine serum albumin (BSA; cat. no. A3059) in 0.1 M phosphate‐buffered saline (PBS, pH 7.4; Sigma‐Aldrich, cat. no. P4417) and 0.1% Triton‐x 100 (Sigma‐Aldrich, cat. no.X‐100). The samples were then incubated overnight at 4°C with the primary antibody, followed by an anti‐mouse HRP‐conjugated secondary antibody. The sections were then treated with the substrate‐chromogen system DAB (Dako; cat. no. K3468). 24‐month‐old brains from TG were used as a positive control. Images were acquired by Leica LMD7 microscope (GmbH, Nussloch, Germany; RRID:SCR_024657).

### 2.7 Immunofluorescence staining

Immunofluorescence (IF) on retinal cryosections was used to identify the localization of the selective microglia marker ionized calcium‐binding adaptor molecule 1 (IBA‐1), Glial fibrillary acidic protein (GFAP), CB_2_, and MAGL, and to quantify them throughout the retinal layers. Sections were washed three times with PBS‐T (PBS 1X (Sigma‐Aldrich, cat. no. P4417) + 0.1% Triton X100 (Sigma‐Aldrich, cat. no. X100) for 10 min). Briefly, non‐specific binding sites were blocked using 5% bovine serum albumin (BSA; Sigma‐Aldrich, cat. no. A3059) in PBS‐T for 1 h at RT. Sections were then incubated overnight at 4°C with primary antibodies diluted in 1% BSA in PBS‐T for detection of microglia (using anti‐IBA‐1), astrocytes/Müller glia (GFAP), CB_2_, MAGL, and acrolein (cat. no. and dilution of the antibodies are reported in Table 1). The following day, sections were washed three times with PBS‐T for 10 min and then incubated with secondary antibodies (anti‐mouse or anti‐rabbit IgG conjugated to a green fluorescent dye; Alexa Fluor 488; Molecular Probes, Invitrogen, Carlsbad, CA, USA) diluted 1:300 in 1% BSA in PBS‐T at 37°C for 2 h. Bisbenzimide nuclear dye (Hoechst; ThermoFisher Scientific, cat. no. H21486) was used to label the nuclei for 2 min at RT. Negative controls were obtained by staining retinal cryosections without the primary antibodies.

### 2.8 Confocal microscopy and image analysis of retinal sections

Images of immunolabeled cryosections were acquired using a Nikon 80i confocal microscope (RRID:SCR_017733). Sections crossing the optic nerve (O.N.) were selected for the analysis and images from the ventral and dorsal retina were acquired and mediated as reported in the result. The same parameters were set up for all the acquisitions. For the final images, 22 planes at a distance of 0.5 μm were acquired using a 40× objective maintaining the same acquisition parameters in terms of gain and offset. The fluorescence intensity of the markers was quantified through ImageJ software and using plot profiles with the corresponding grayscale intensities (range 0–50). The values were normalized on the selected area. The quantification of microglial cells was performed by counting IBA‐1 positive cells across the entire central sections. The results are presented as the number of IBA‐1 positive cells (n° IBA‐1+ cells). The final representative images reported in Figures 2 and 5 were acquired using a Leica TCS SP5 confocal microscope (Leica; RRID:SCR_020233).

The morphological analyses were performed on sections crossing the optic nerve from the dorsal to the ventral direction. The analysis of central sections was necessary to assess the thickness of the retinal nuclear layers, a well‐known hallmark of retinal degeneration (Parisi et al., 2001; van de Kreeke et al., 2019). The analysis was carried out by staining the sections with the bisbenzimide nuclear dye to measure the thickness for both the photoreceptors and inner nuclear layers, evaluated as the ratio of outer nuclear layer (ONL)/total retina thickness and inner nuclear layer (INL)/total retina thickness. Measurements were performed by Image J software, and images were acquired by fluorescence microscopy (Nikon, eclipse 80i; RRID:SCR_017733) with a 20× objective.

### 2.9 Ultra‐performance liquid chromatography–tandem mass spectrometry (UPLC‐MS/MS)

The analysis of the eCBs profile was performed in 3, 6, and 12 months retinas using a new UPLC‐MS/MS procedure that we reported elsewhere (Fanti et al., 2023) with slight modifications. Briefly, samples underwent homogenization and extraction with MeOH using a Precellys 24 tissue homogenizer (Bertin, Montigny‐le‐Bretonneux, France; RRID:SCR_022979). Internal standards were spiked into the extraction solvent at a final concentration of 5 ng/mL to standardize the analysis. Subsequently, a clean‐up step employing OMIX C18 micro tips (Agilent Technologies, cat. no. AGA57003MBK) preceded UPLC‐MS/MS analysis.

Instrumentation included a Qtrap 4500 mass spectrometer (Sciex, cat. no. 904‐0000) coupled with a Waters UPLC Acquity H‐Class (Waters, Milford, Massachusetts, USA) Waters SQD2 LC/MS system (RRID:SCR_022217). Electrospray ionization operated in positive mode (ESI+) for the acquisition of both AEA and 2‐AG. Analytes were separated using a Waters Acquity BEH C18 column (100 × 2.1 mm, packed with 1.8 μm particles). Data analysis was performed using Analyst 1.7.3 software Analyst®TF Software (RRID:SCR_015785), with chromatographic peak integration and quantification of target analytes performed using MultiQuant 3.0.3 software, both provided by Sciex.

### 2.10 Glutamate quantification

Levels of glutamate were quantified in retinal samples of 12‐month‐old TG and WT mice using the Glutamate‐Glo™ assay (Promega, cat. no. J7021). Briefly, retinal samples were lysed in a Homogenizing Buffer containing 50 mM Tris (pH 7.5) pre‐mixed with 0.6 N HCl (Inactivation solution; 8:1 v/v) at 6 mg of tissue/mL. Immediately after homogenization, 1 M Tris Base was added (the same volume of Inactivation solution), the samples were centrifuged (17 968 *g* for 20 min at 4°C) and the surnatant was collected and stored at −80°C until use. The assay was then performed according to manufacturer instructions, using 50 μL of the homogenized tissue and standard glutamate references (from 50 to 1.6 μM) in 96 white multiwell (Greiner cat. no. 655083). 50 μL of Glutamate Detection Regent was added to each well and luminescence was recorded after 1 h of incubation at room temperature, using a TECAN Magellan Pro v7.4 microplate reader (Promega; cat. no. 30172786). Data were expressed as μmol/g retina.

### 2.11 Statistical analysis

The sample size was estimated based on our previous experience with Tg2576 mice (Maccarrone et al., 2018) and on previous studies with retinas of the same model (Liu et al., 2009; Morello et al., 2023). All results are reported as mean ± standard error of the mean (SEM). Data were screened for normality using the Saphiro‐Wilk test; data were considered normally distributed when *p* was >0.05. Afterwards, a two‐tailed Student's *t*‐test or Mann–Whitney test was used to compare TG and WT mice. Grubbs' test was used to identify the outliers. Linear regression analysis was performed on CB_2_ (independent) and APP and other ECS proteins (dependent), setting *p* < 0.05 for statistical significance. Measurements were plotted and analyzed using GraphPad Prism version 9 (RRID:SCR_002798) or Sigma Plot 12.0 (RRID:SCR_003210). No blinding was performed.

## 3 RESULTS

### 3.1 Twelve‐month‐old Tg2576 mice do not show β‐amyloid plaques in the hippocampus and in the retina

**FIGURE 1:**
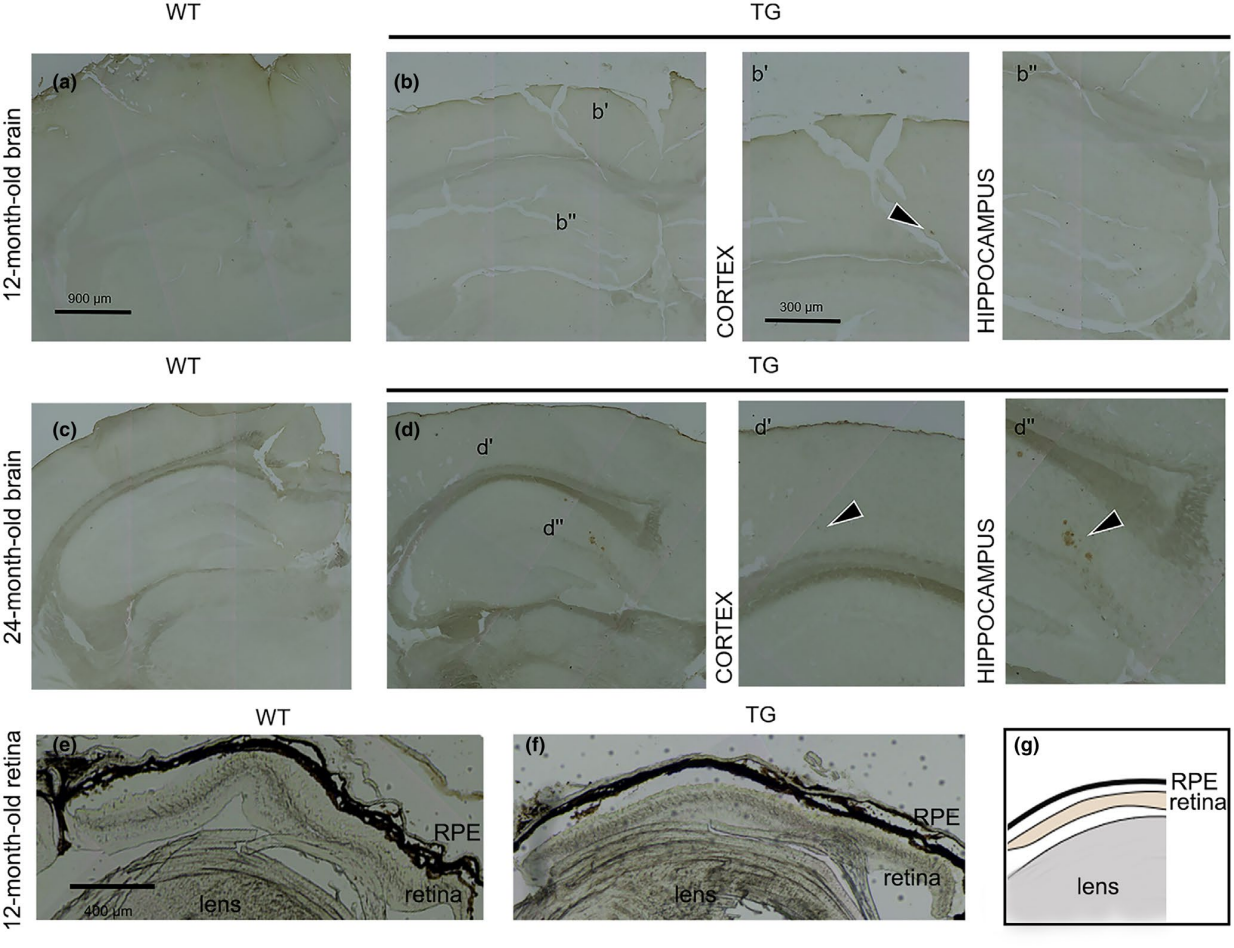
Immunohistochemistry of β‐amyloid in WT and TG brains. Representative brain cryosections of WT and TG mice stained with anti‐β‐amyloid immunohistochemistry (IHC). (a) 12‐month‐old WT, (b) 12‐month‐old TG, (b’) high magnification of cortex, (b”) high magnification of hippocampus. (c) 24‐month‐old WT, (d) 24‐month‐old TG, (d’) high magnification of cortex, (d”) high magnification of hippocampus. Scale bars: 900 and 300 μm. The black arrows indicate the β‐amyloid plaques. (e, f) Representative eye cryosections stained with anti‐β‐amyloid IHC in WT and TG mice respectively. Scale bar: 400 μm. (g) Schematic representation of ocular tissues visible in images e and f.

To investigate early retinal events occurring in AD, we selected 12‐month‐old Tg2576 mice, which still do not exhibit β‐amyloid plaques in the hippocampus at this time point (Snellman et al., 2013). To confirm the suitability of the selected time point, anti‐ β‐amyloid IHC was performed on brain cryosections to evaluate the accumulation of β‐amyloid plaques in the brain of 12‐month‐old TG mice (Figure 1). We did not detect any β‐amyloid plaques in the hippocampus of TG mice (Figure 1a,b,b”), while sporadic and small β‐amyloid plaques could be detected in the cortex of TG mice only (Figure 1b,b’). As additional controls, we used also 24‐month‐old WT and TG (Figure 1c,d) mice, that showed the development of β‐amyloid plaques both in cortex (Figure 1d,d’) and hippocampus (Figure 1d,d”). We also investigated whether β‐amyloid plaques could occur in the retina of 12‐month‐old mice, by performing anti‐β‐amyloid IHC on retinal cryosections (Figure 1e–g). However, no β‐amyloid plaques were detected.

### 3.2 Tg2576 retinas show reactive gliosis and oxidative stress burden without neuronal degeneration

**FIGURE 2:**
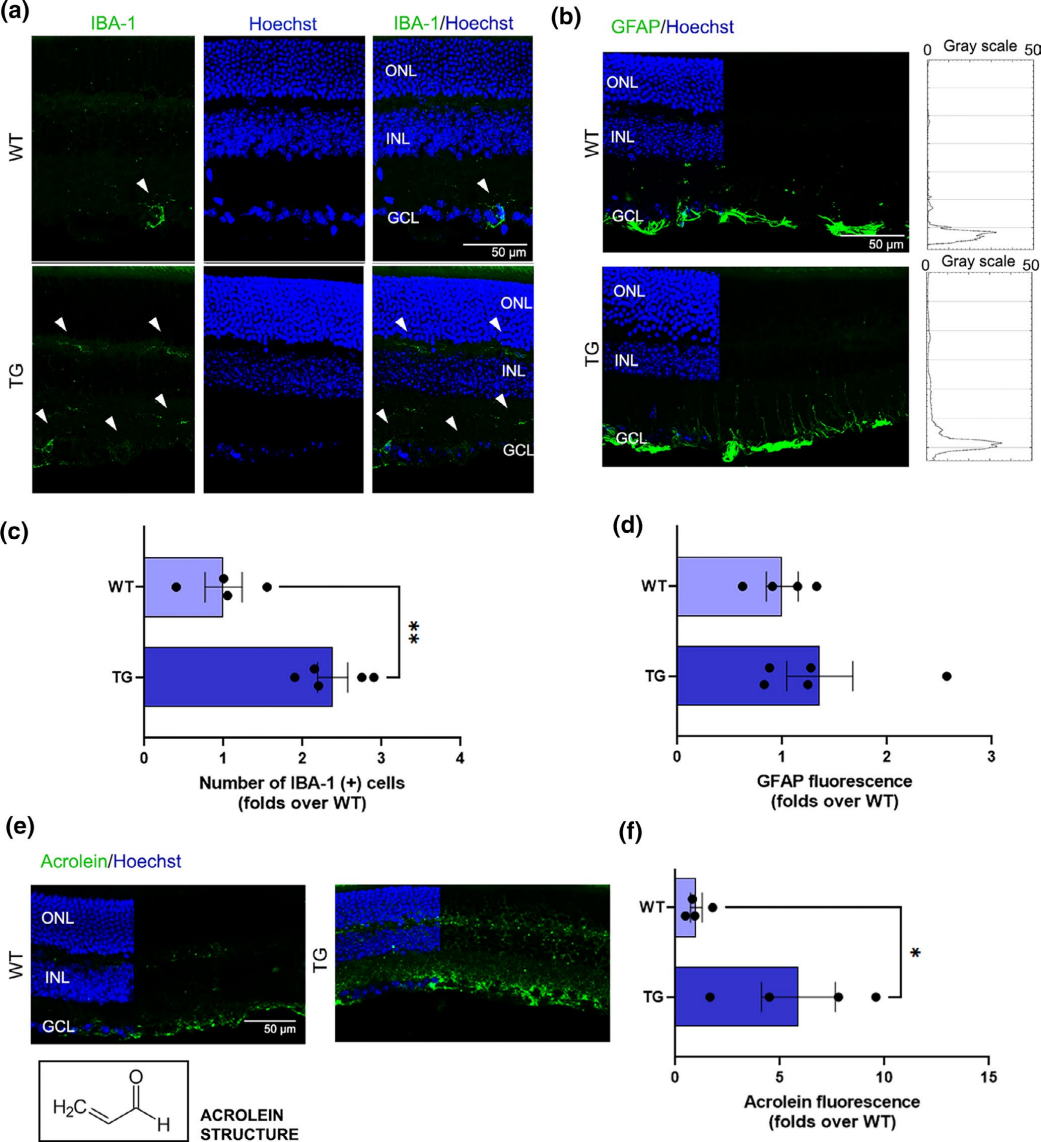
Analysis of gliosis. (a) Representative confocal images of WT and TG retinal cryosections immunostained with anti‐IBA‐1 to identify microglia cells. The white arrows indicate the IBA‐1 positive cells. Scale bar: 50 μm. (b) Representative confocal images of WT and TG retinal cryosections immunostained with anti‐GFAP to identify astrocytes and Müller cells reactivity, with corresponding plot profile graphs showing GFAP fluorescence throughout the retinal layers. Scale bar: 50 μm. (c) Quantification of IBA‐1 (+) cells counted on retinal cryosections of all experimental groups. Data are shown as mean ± SEM. The black dots indicate individual sample values (*n* = 4–5). (d) Quantification of GFAP fluorescence intensity on retinal cryosections of all experimental groups. Data are shown as mean ± SEM. The black dots indicate individual sample values(*n* = 4–5). (e) Representative confocal images of WT and TG retinal cryosections immunostained with anti‐Acrolein to investigate oxidative stress. The black box highlights the Acrolein chemical structure. Scale bar: 50 μm. (e) Quantification of Acrolein fluorescence intensity on retinal cryosections of all experimental groups. Data are shown as mean ± SEM. The black dots indicate individual sample values. Scale bar: 50 μm. Details of the statistics are reported in Table S1. For abbreviations see the abbreviations list.

To investigate retinal neuroinflammation, we explored the possible development of retinal gliosis. To gain this purpose IBA‐1 (Figure 2a) and GFAP (Figure 2b) immunostainings were performed on retinal cryosections of both experimental groups. Microglia number was calculated by counting IBA‐1 positive cells (Figure 2c). Interestingly, we found a significant increase in the number of IBA‐1‐positive microglia in TG compared to WT mice (2.5 folds over WT; *p* = 0.002). We also investigated the possible reactivity of astrocytes and Müller cells, via anti‐GFAP immunostaining (Figure 2b). Although plot profile graphs showed a trend towards an increase in GFAP staining in TG compared to WT at the inner retina, densitometric analysis of the images showed no statistically significant differences between groups (Figure 2d). Hence, early neuroinflammatory events occurring in the retina of TG mice mostly involved microglia rather than macroglia cells.

To further investigate the pathological hallmarks occurring in the retina of TG mice, we investigated the possible increase of acrolein (Figure 2e,f), a well‐known by‐product of lipid peroxidation and a marker of retinal stress, through the quantification of anti‐acrolein staining in retinal cryosections, as previously reported (Albarracin et al., 2013; Tisi et al., 2019). A significant increase in acrolein fluorescence intensity was found in TG versus WT retinas (Figure 2f), indicating a condition of oxidative stress burden in TG retinas.

**FIGURE 3:**
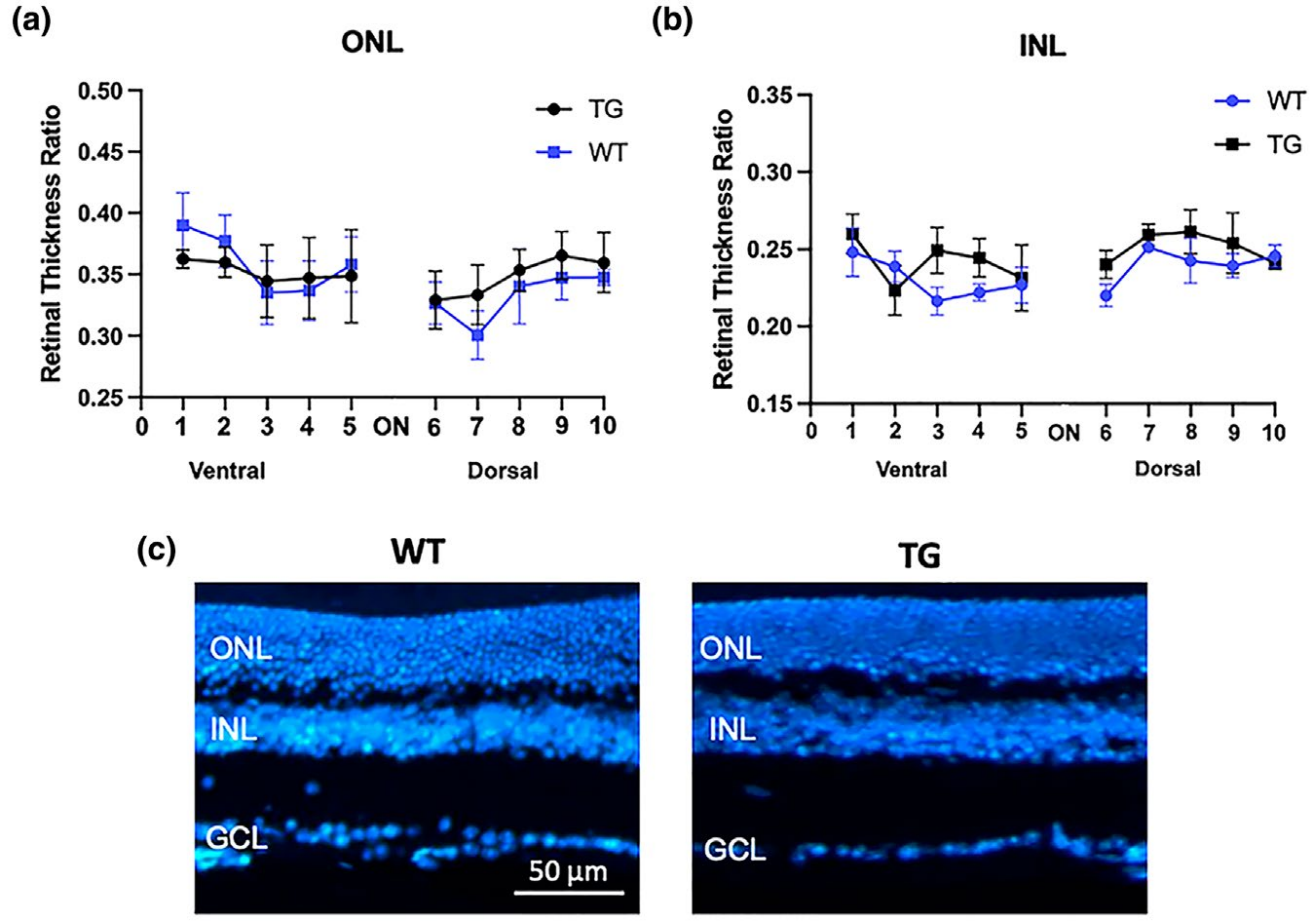
Retinal thickness. Analysis of ONL (a) and INL (b) thickness from ventral to dorsal direction on retinal cryosections crossing the optic nerve (ON). (c) Representative fluorescence images of WT and TG retinal cryosections stained with bisbenzimide nuclear dye (blue). Measurements are expressed as the ratio of ONL/total retina thickness and INL/total retinal thickness calculated along the vertical meridian. Scale bar: 50 μm. Data are shown as mean ± SEM (*n* = 4–5). For abbreviations see the abbreviations list. Details of the statistical analyses are reported in Table S1.

Next, we performed morphological analysis to investigate the possible thinning of retinal nuclear layers, a known feature of retinal degeneration (Tisi et al., 2019). No significant difference in thickness was found in either ONL (Figure 3a) or INL (Figure 3b) between the two experimental groups, indicating that neuroinflammatory events occur in the absence of retinal degeneration in AD‐like mice. Figure 3c shows representative images of retinal sections stained with bisbenzimide nuclear dye (blue).

### 3.3 CB_2_ and MAGL are up‐regulated in the retina of Tg2576 mice

In order to investigate whether ECS alterations develop in the retina of AD‐like mice, we quantified the major ECS receptors and metabolic enzymes.

**FIGURE 4:**
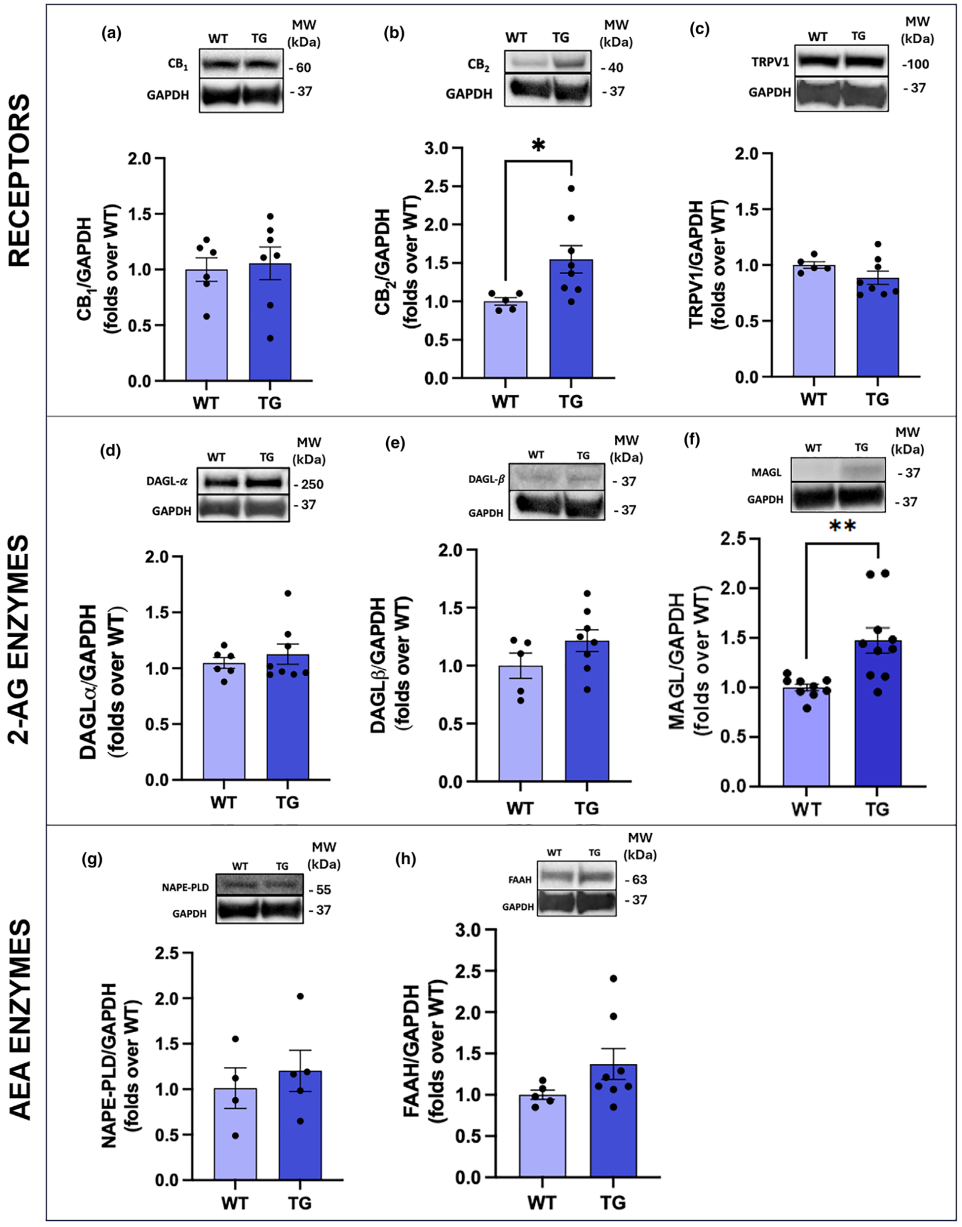
Retinal expression of ECS components. Western blot analysis of CB_1_ (a), CB_2_ (b), TRPV1 (c), DAGLα (d), DAGLβ (e), MAGL (f), NAPE‐PLD (g), and FAAH (h) in the retinas of the two experimental groups. Molecular weights (MW) are shown on the right‐hand side. Data are shown as mean ± SEM (*n* = 4–10). The black dots indicate individual samples. Details of the statistics are reported in Table S1. Original whole western blot bands are reported in Figures S1–S8. Some proteins were probed on the same membranes and, therefore, the GAPDH housekeeping band is similar within each respective pair of figures (Table S4). For abbreviations see the abbreviations list.

We first investigated the expression of the main eCBs‐binding receptors (CB_1_, CB_2_, TRPV1) by western blot in the retinas of TG and WT mice (Figure 4a–c). We did not observe any differences between TG and WT in CB_1_ (Figure 4a) and TRPV1 (Figure 4c) expression. Instead, the expression of CB_2_ was significantly increased in TG compared to WT (1.5 folds over WT; Figure 4b).

Then, we investigated the expression of DAGLα/β and MAGL, the enzymes involved in the biosynthesis and degradation of 2‐AG, respectively (Figure 4d–f). Despite a trend towards an increase of protein levels in TG, neither DAGLα (Figure 4d) nor DAGLβ (Figure 4e) showed a statistically significant increase, whereas the expression of MAGL increased in a statistically significant manner (1.5 folds over WT; Figure 4f).

Finally, we investigated the protein levels of the biosynthetic and hydrolytic enzymes of AEA, NAPE‐PLD, and FAAH, respectively. We did not observe any significant differences in the expression of NAPE‐PLD (Figure 4g) and FAAH (Figure 4h).

**FIGURE 5:**
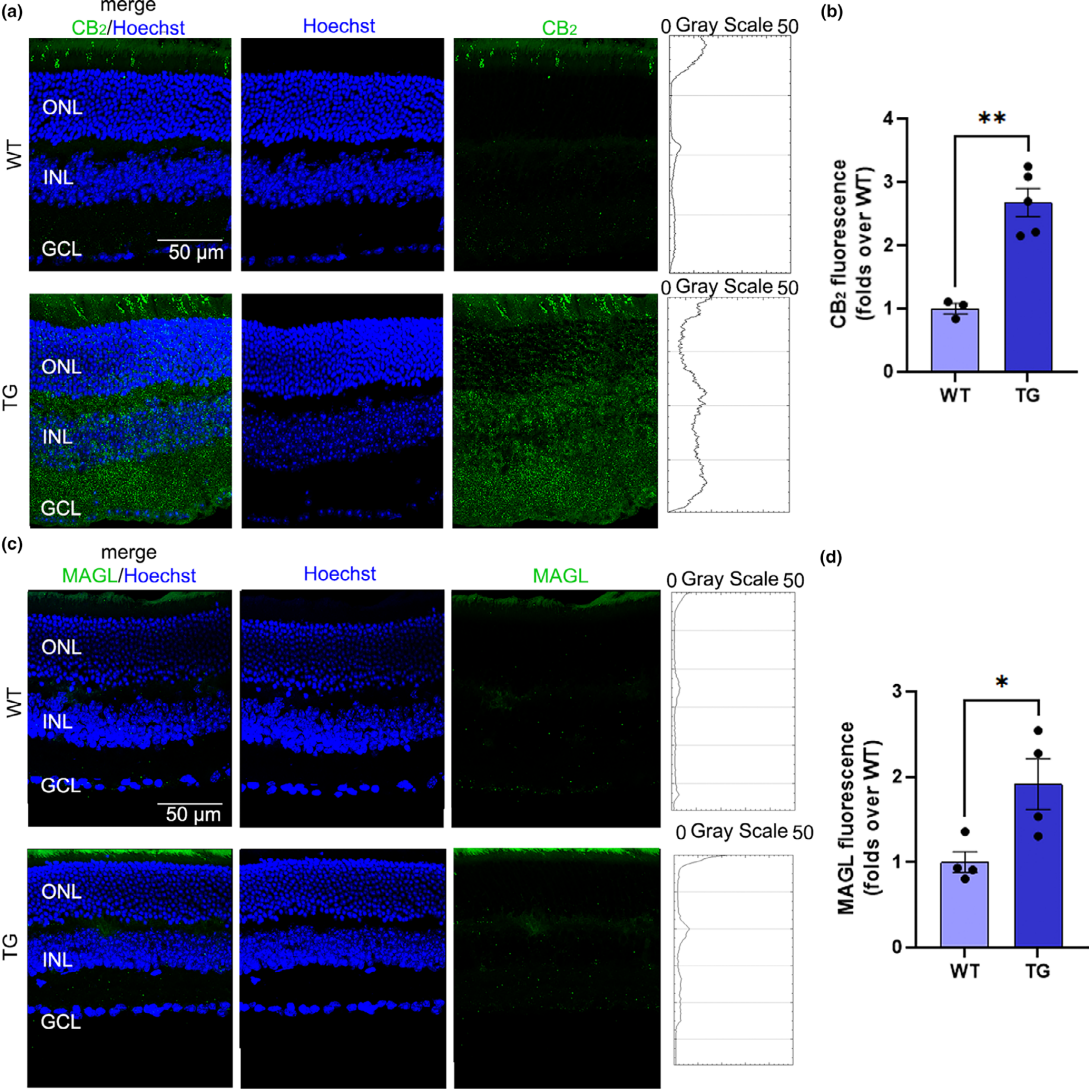
CB_2_ and MAGL immunostaining. (a) Confocal images of cryosections immunolabeled for CB_2_ (green) from WT and TG retinas with relative plot profile graphs showing fluorescence intensity through the retinal layers. (b) Quantification of CB_2_ fluorescence intensity. (c) Confocal images of cryosections immunolabeled for MAGL (green) from WT and TG retinas with relative plot profile graphs showing fluorescence intensity through the retinal layers. (d) Quantification of MAGL fluorescence intensity. Scale bar: 50 μm. Data are shown as mean ± SEM (*n* = 3–5). The black dots indicate individual samples. Details of the statistics are reported in Table S1. For abbreviations see the abbreviations list.

To further investigate the up‐regulation of CB_2_ and MAGL in TG retinas, their expression and localization were also assessed on retinal cryosections through immunofluorescence (Figure 5a,c). The immunostaining of CB_2_ and MAGL in the retina was consistent with a previous study (Bouskila et al., 2016). The signal of both proteins was increased throughout the retinal layers, as shown in the plot profile graphs (Figure 5a,c), with MAGL resulting in a lower expression in both experimental groups compared to CB_2_. Quantitative analysis of fluorescence intensity demonstrated that CB_2_ (Figure 5b) and MAGL (Figure 5d) expression was significantly up‐regulated in TG compared to WT retinas, confirming western blot data. A retinal cryosection incubated with a secondary antibody only was used as a negative control (Figure S9).

### 3.4 2‐AG is down‐regulated in the AD‐like mouse retina

**FIGURE 6:**
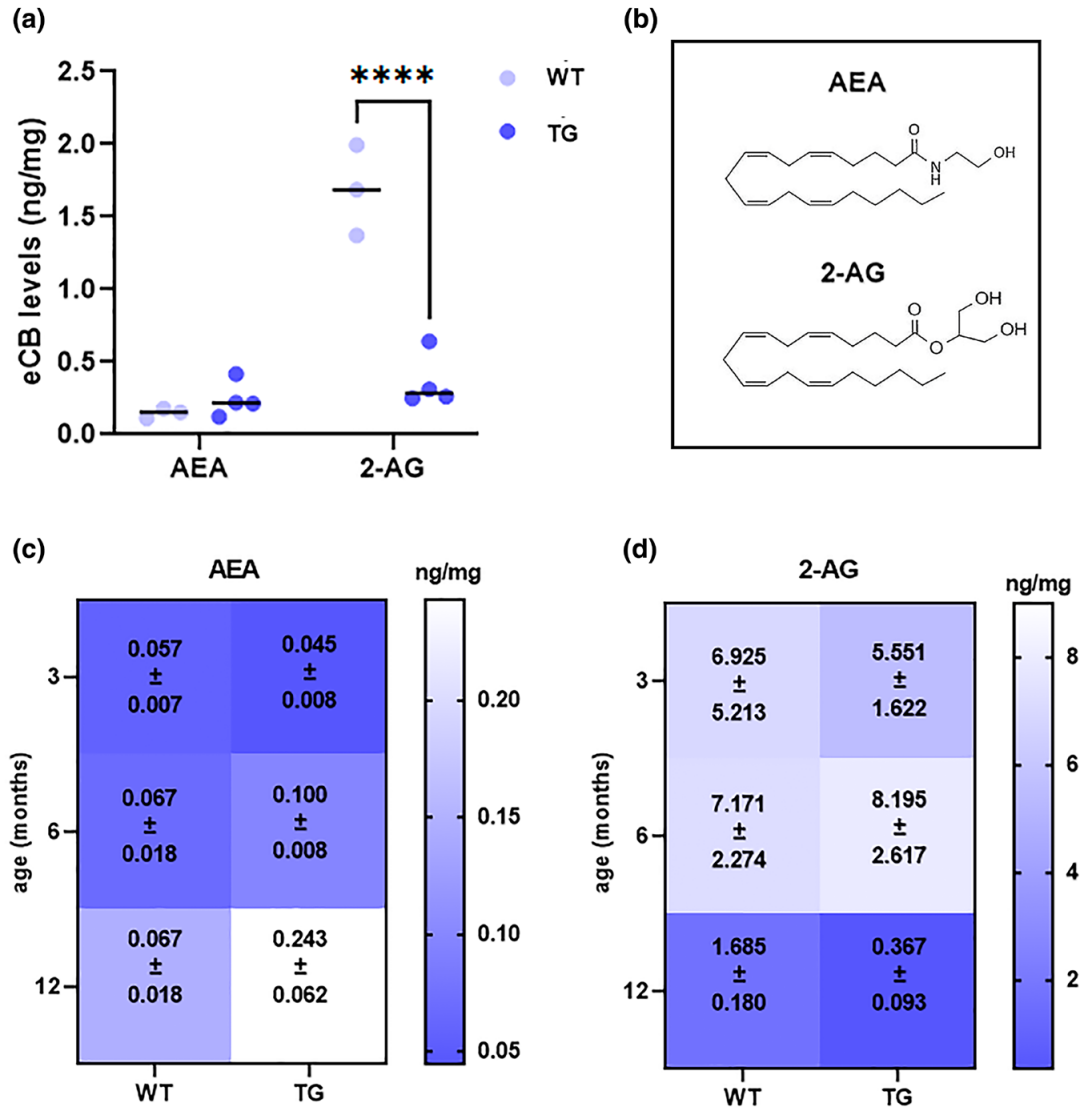
Endogenous levels of AEA and 2‐AG in the retinas of WT and TG mice. (a) The figure shows UPLC‐MS/MS analysis of AEA and 2‐AG in 12‐month‐old TG and WT mice. The dots indicate individual samples (*n* = 3–4). (b) Chemical structures of AEA and 2‐AG. (c) Heatmap showing AEA levels in 3‐, 6‐ and 12‐month‐old retinas of WT and TG; (d) heatmap showing 2‐AG levels in 3‐, 6‐, and 12‐month‐old retinas of WT and TG. The numbers on the heatmaps represent mean ± SEM (*n* = 3–4). Details of the statistics are reported in Table S1. For abbreviations see the abbreviations list.

After investigating eCBs‐binding receptors and metabolic enzymes, the retinas of TG and WT mice were screened for quantification of the two major eCBs (AEA and 2‐AG; whose chemical structures have been reported in Figure 6b) via UPLC‐MS/MS (Figure 6a).

Consistently with the increase of MAGL protein levels, UPLC‐MS/MS revealed a significant reduction of 2‐AG in TG retinas (~0.34 ng/mg) compared to WT (~1.7 ng/mg). Instead, a trend towards increase was found for AEA (WT: ~0.15 ng/mg; TG: ~0.24 ng/mg), but the difference was not statistically significant.

Additionally, to investigate the earliest time point of eCB modifications in the retinas of TG mice, we performed a time‐course analysis of eCB levels also in the retinas of 3‐ and 6‐month‐old mice. The results were graphed alongside the 12‐month‐old data as a heatmap (Figure 6c,d). An age‐related trend was observed with a tendency towards increase for AEA and decrease for 2‐AG in older retinas compared to the younger ones in either WT and TG; however, statistically significant differences between WT and TG were present for 2‐AG in 12‐month‐old retinas only (Statistical results are reported in Table S1). This data indicate that the 12 months are a critical time point for the modifications of the eCB system in the TG retina and further supports the suitability of the selected time point for the investigation for the eCB signaling in the Tg2576 model.

Since 2‐AG plays a modulatory role on retinal synapses (Bouchard et al., 2016), we wondered whether its reduction in TG retinas was associated with glutamate alterations, likely leading to retinal excitotoxicity. However, glutamate quantification showed no statistically significant differences between WT and TG retinas (Figure S11).

### 3.5 Linear regression analysis for individual animals

To better understand the eCB‐associated metabolic events in the retina of AD‐like mice, we also performed linear regression analysis for individual animals between the ECS receptors/enzymes and: (i) CB_2_ (Table S2), (ii) MAGL (Table S3), and (iii) APP (only in TG retinas; Table 2; Figure S10).

**TABLE 2:** Linear regression analysis between APP and ECS components in TG retinas.

	APP			
	*f*(x)	*r*	*R* ^2^	*p*‐value
CB_1_	*y* = 1.330–0.038 *x*	0.200	0.040	0.667
CB_2_	*y* = 0.290 + 0.724 *x*	0.789	0.622	**0.020**
TRPV1	*y* = 0.654 + 0.334 *x*	0.400	0.165	0.318
DAGLα	*y* = 1.026 + 0.014 *x*	0.115	0.001	0.787
DAGL𝛽	*y* = 1.726–0.073 *x*	0.568	0.323	0.142
MAGL	*y* = 1.382–0.357 *x*	–0.7843	0.615	**0.037**
NAPE‐PLD	*y* = 0.107 + 0.140 *x*	0.609	0.370	0.276
FAAH	*y* = 0.824 + 0.079 *x*	0.306	0.009	0.461

*Note*: Linear regression analysis was performed for individual animals to investigate the existence of correlations between APP and the ECS receptors/enzymes in TG retinas. The table shows the equation, *r*, *R* ^2^ and *p*‐value for each ECS element. The *p*‐values<0.05 are highlighted in bold.For abbreviations see the abbreviations list.

**FIGURE 7:**
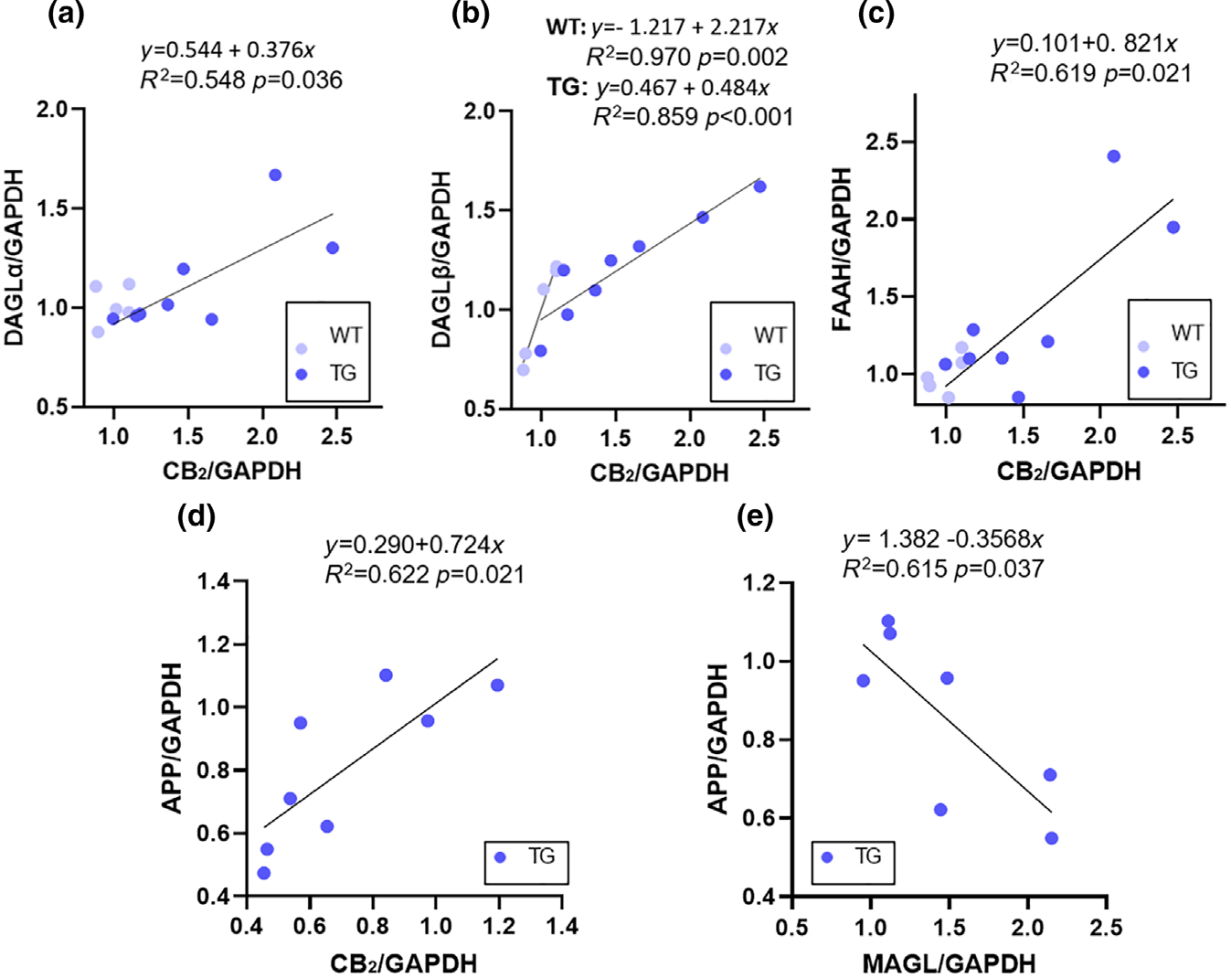
Scatter plot of ECS receptors/enzymes and APP levels in individual animals. (a) DAGLα versus CB_2_, (b) DAGLβ versus CB_2_, (c) FAAH versus CB_2_, (d) CB_2_ versus APP, (e) MAGL versus APP. Data points indicate individual animals; the black line indicates the linear regression line. For abbreviations see the abbreviations list.

Linear regression analysis showed that increased levels of CB_2_ positively correlated with DAGLα/β (Figure 7a,b) and FAAH (Figure 7c) levels in TG retinas, while no significant correlations were found with the other ECS components (Table S2). Moreover, MAGL protein levels did not show any statistically significant correlations with the other ECS components (Table S3). Of note, APP showed a significant correlation with both CB_2_ (Figure 7d) and MAGL (Figure 7e), which was positive with the former and negative with the latter.

## 4 DISCUSSION

The relationship between neuroinflammation and ECS in AD is becoming increasingly evident thanks to several studies indicating a pivotal role of eCBs and their signaling in the control of inflammatory events related to AD pathogenesis (Crehan et al., 2012). Moreover, the concept that the retina may serve as a window to study brain‐related processes (Crehan et al., 2012) calls for the need to delve deeper into the mechanisms and molecular underpinnings of AD. On this basis, in the present study, we investigated the extent of gliosis events and the possible alterations of the ECS in the retina of Tg2576 mice, which exhibit pathological features similarly to human AD because of the overexpression of the mutated human APP. First, our study demonstrated the presence of retinal neuroinflammation in 12‐month‐old TG mice. Particularly, gliosis in TG retinas primarily involved microglia cells rather than astrocytes/Müller cells. The reactivity of microglia is in line with previous literature data, as microglial activation and microgliosis have been well documented in AD (Grimaldi et al., 2018; Salobrar‐García et al., 2020) and are consistent with the broader inflammatory response seen in the brain (Crehan et al., 2012). Importantly, our data indicate that microglia reactivity in the retina is an early event in AD‐like mice, occurring well before the onset of evident retinal/brain pathological signatures. Particularly, 12‐month‐old TG mice did not show: (i) retinal degeneration (Figure 3), (ii) development of hippocampal and retinal β‐amyloid plaques (Figure 1), and (iii) glutamate excitotoxicity (Figure S11). Instead, increased acrolein levels were observed in the retina of TG versus WT mice (Figure 2e,f), indicating a condition of oxidative stress burden. It seems noteworthy that a previous study demonstrated retinal thickness reduction in Tg2576 mice starting from 14 months of age only (Liu et al., 2009). Hence, our data further support that this mouse model at 12 months may be in an early phase of the disease. In line with this, retinal microglia reactivity observed in TG mice claims a possible pivotal role of this cytotype at the initial stages of AD, possibly associated with pathological features different from β‐amyloid plaques accumulation or neuronal degeneration, such as oxidative stress. Accordingly, the concept that microglia and neuroinflammation may play an initiating role in AD is supported by an increasing number of studies and is revolutionizing the generally accepted amyloid cascade hypothesis (Crehan et al., 2012). In this complex scenario, our findings further support microglia as major players in AD pathogenesis and demonstrate their involvement in AD‐associated retinal manifestations of Tg2576 mice, suggesting that similar events may occur in the human retina as well.

In the context of the neuroinflammatory processes underlying AD, the ECS is emerging as a key molecular network, regulating inflammation and a plethora of other neurological functions (Maccarrone et al., 2023). Hence, accumulated evidence indicates that ECS dysregulation has important implications in AD pathogenesis (Cristino et al., 2020). In the present study, we show for the first time that an imbalance of the ECS develops in the retina of AD‐like mice as well and is likely to be an early event of the pathology, preceding retinal remodeling and hippocampal β‐amyloid plaques deposition. Particularly, we found a significant up‐regulation of CB_2_ receptor in 12‐month‐old TG retinas, which is in line with the increase of microglial cells. Indeed, previous literature has extensively documented that the up‐regulation of CB_2_ is linked to excessive microglial cell proliferation in various neuroinflammatory diseases. Notably, CB_2_ was abundantly expressed in microglia cells in the brains of AD patients. Likewise, AD mouse models also displayed a substantial elevation in CB_2_ levels within microglia (Benito et al., 2003, 2008). However, double labeling of CB_2_ and Hoechst as a nuclear dye revealed that CB_2_ up‐regulation did not occur in a specific retinal location and involved all retinal layers (Figure 5), indicating that multiple cytotypes are responsible for the overall increase of CB_2_ levels. Importantly, we also demonstrated a significant down‐regulation of 2‐AG levels in TG retinas compared to WT at 12 months only, indicating that this is the ideal time point to study eCB alterations in the retina of the Tg2576 mouse. This result was accompanied by the up‐regulation of MAGL protein levels, the primary enzyme for 2‐AG degradation. Notably, the engagement of 2‐AG/MAGL axis has previously been described in AD brains (Chen, 2022). In particular, high levels of MAGL have been documented in the brain of authentic patients and animal models of AD (Farooqui et al., 1988; Syal et al., 2020). Consistently, reduced 2‐AG levels have been found in the brain of AD‐like mice (Maroof et al., 2014). It should be recalled that 2‐AG hydrolysis releases AA, which may serve as a substrate for the biosynthesis of prostaglandins and leukotrienes, two major families of pro‐inflammatory mediators (Chen, 2022; Nomura et al., 2011). These latter molecules in turn may contribute to broadening the inflammatory processes of AD in both the brain and the retina. Moreover, since 2‐AG displays anti‐inflammatory and neuroprotective properties, its degradative enzyme MAGL has been proposed as a therapeutic target in AD to enhance 2‐AG tone, and hence neuroprotection, with promising results in preclinical models (Chen et al., 2012; Hashem et al., 2021). Of note, a previous study in AD‐like mice lacking CB_2_ demonstrated that neuroprotection induced by the inhibition of MAGL is independent of CB_2_ expression (Zhang & Chen, 2018). This could explain the opposing trends of CB_2_ and MAGL in relationship to APP in individual animals, shown herein (Figure 7d,e), further suggesting that multiple ECS‐related mechanisms may contribute to retinal manifestations in the Tg2576 model. For instance, it is known that the ECS plays an important role in the regulation of synaptic function (Bouchard et al., 2016). In line with this, we hypothesized that the eCB alterations observed here may be involved in retinal functional deficits. One possibility could be the glia‐neurons “dialog” in the context of neurotransmitters release/metabolism (Czapski & Strosznajder, 2021). This would be in line with the increased microglia content found in TG retinas since microglia play a pivotal role in the control of glutamatergic synapses (Basilico et al., 2019, 2022) and glutamate receptors' dysfunction in microglia has been previously associated with AD (Noda, 2016). Moreover, it is known that 2‐AG‐mediated CB_1_ activation regulates glutamate release at the synapse (Wilson & Nicoll, 2002), and, therefore, the reduction of 2‐AG content observed here would further suggest a dysregulation of glutamate neurotransmission. On this basis, we quantified glutamate levels in the retinas of TG and WT mice but we failed to find any differences (Figure S11), suggesting that glutamate neurotransmission may not be implicated in the retinal pathology of Tg2576 mice at 12 months. Yet, it should be noted that a conclusive statement on the implications of the ECS dysregulation in TG retinal function/synaptic activity awaits to be further supported. For instance, the quantification of other retinal neurotransmitters or electroretinography assessment would be needed to better clarify this issue.

Overall, our study demonstrated an imbalance of eCBs signaling because of increased expression of CB_2_ and MAGL, as well as to reduced content of 2‐AG, in the retina of AD‐like mice well before the development of hippocampal β‐amyloid plaques. Similar molecular results have been previously demonstrated in AD brains of humans and mouse models. Additionally, we showed an increased number of microglial cells in the retinas of Tg2576 mice, indicating the presence of inflammatory processes similar to those observed in the brain, alongside an increased oxidative stress. Hence, the concept that the retina may indeed serve as a “window to the brain” underscores the significance of our findings, which further support the potential of the retina as a valuable tool for investigating and understanding neuroinflammatory conditions of the brain.

## AUTHOR CONTRIBUTIONS

Conceptualization: Annamaria Tisi, Mauro Maccarrone; funding acquisition: Annamaria Tisi, Sergio Oddi, Mauro Maccarrone; methodology: Annamaria Tisi, Lucia Scipioni, Giulia Carozza, Lucia Di Re, Giacomo Cimino, Camilla Di Meo, Sakthimala Palaniappan, Francesco Della Valle, Federico Fanti, Giacomo Giacovazzo; supervision: Mauro Maccarrone; formal analysis: Annamaria Tisi, writing—original draft preparation: Annamaria Tisi; writing—review and editing: Annamaria Tisi, Lucia Scipioni, Giulia Carozza, Lucia Di Re, Giacomo Cimino, Camilla Di Meo, Sakthimala Palaniappan, Francesco Della Valle, Federico Fanti, Giacomo Giacovazzo, Dario Compagnone, Rita Maccarone, Sergio Oddi, Mauro Maccarrone.

## CONFLICT OF INTEREST STATEMENT

The authors declare no conflicts of interest.

## Supporting information


Data S1.

## Data Availability

Data available on request from the authors.
