# Supplementary material for: Alterations of endocannabinoid signaling and microglia reactivity in the retinas of AD‐like mice precede the onset of hippocampal β‐amyloid plaques
Source: J Neurochem. 2024 Nov 18;169(2):e16256. doi: 10.1111/jnc.16256 (PMC11808635; doi:10.1111/jnc.16256)

Supplementary material

Alterations of endocannabinoid signalling and microglia reactivity in the retinas of AD-like mice precede the onset of hippocampal β-amyloid plaques

Annamaria Tisi[1] *, Lucia Scipioni[1,2], Giulia Carozza[1], Lucia Di Re[1], Giacomo Cimino[1], Camilla Di Meo[1,4], Sakthimala Palaniappan[1], Francesco Della Valle[3], Federico Fanti[3], Giacomo Giacovazzo[2,4], Dario Compagnone[3], Rita Maccarone[1], Sergio Oddi[2,4], Mauro Maccarrone[1,2] *

[1] Department of Biotechnological and Applied Clinical Sciences, University of L'Aquila, 67100 L'Aquila, Italy.

[2] Laboratory of Lipid Neurochemistry, European Center for Brain Research (CERC), Santa Lucia Foundation IRCCS, 00143 Rome, Italy.

[3] University of Teramo, Department of Bioscience and Technology for Food, Agriculture and Environment, 64100, Teramo, Italy.

[4]University of Teramo, Department of Veterinary Medicine, 64100, Teramo, Italy.

*Corresponding authors

Authors' e-mail addresses:

annamaria.tisi@univaq.it, lucia.scipioni@graduate.univaq.it,
giulia.carozza@graduate.univaq.it, lucia.dire@gmail.com,
giacomo.cimino@graduate.univaq.it, cdimeo@unite.it,
sakthimala.palaniappan@graduate.univaq.it, fdellavalle@unite.it, ffanti@unite.it,
giacomogiacovazzo@virgilio.it, dcompagnone@unite.it, rita.maccarone@univaq.it,
soddi@unite.it, mauro.maccarrone@unvaq.it

Corresponding authors details:

Annamaria Tisi: annamaria.tisi@univaq.it
University of L'Aquila
Department of Biotechnological and Applied Clinical Sciences
Vetoio Street, Coppito 2, 67100, L'Aquila, Italy
+39 0862433558
Mauro Maccarrone: mauro.maccarrone@univaq.it
University of L'Aquila
Department of Biotechnological and Applied Clinical Sciences
Vetoio Street, Coppito 2, 67100, L'Aquila, Italy
+39 0862433547

Supplementary Table 1. Statistical analysis performed for data of Figures 2, 3, 4, 5 and 6. All data were screened for normal distribution through the Saphiro-Wilk test and data with p>0.05 were considered normally distributed and were analyzed throught the parametric Student's t-test (degrees of freedom, t value and p value are reported in the table). Data with with p<0.05 according to the Saphiro-Wilk test, were not considered normally distributed and were analyzed through the non-parametric Mann-Whitney test.

Figure	Statistical test	degrees of freedom	t value	p value	Saphiro-Wilk test (p value)
**FIGURE 2**					
2 c	Student's *t*-test	7	-4.620	0.002	0.578
2 d	Student's *t*-test	7	-0.935	0.381	0.078
2 f	Student's *t*-test	6	-2.738	0.034	0.897
**FIGURE 3**					
3 a (field 1)	Student's *t*-test	7	-1.104	0.306	0.666
3 a (field 2)	Student's *t*-test	7	-0.741	0.483	0.219
3 a (field 3)	Student's *t*-test	7	0.234	0.821	0.671
3 a (field 4)	Student's *t*-test	7	0.241	0.816	0.106
3 a (field 5)	Student's *t*-test	6	-0.215	0.837	0.124
3a (field 6)	Student's *t*-test	7	0.0806	0.938	0.614
3a (field 7)	Student's *t*-test	6	0.477	0.650	0.644
3a (field 8)	Student's *t*-test	7	0.398	0.702	0.185
3a (field 9)	Student's *t*-test	7	0.661	0.530	0.908
3a (field 10)	Student's *t*-test	7	0.419	0.688	0.843
3b (field 1)	Student's *t*-test	7	-0.589	0.574	0.788
3b (field 2)	Student's *t*-test	6	0.811	0.448	0.609
3b (field 3)	Student's *t*-test	7	-1.765	0.121	0.838
3b (field 4)	Student's *t*-test	7	-1.523	0.172	0.928
3b (field 5)	Student's *t*-test	7	-0.172	0.868	0.572
3b (field 6)	Student's *t*-test	7	-1.664	0.140	0.592
3b (field 7)	Student's *t*-test	7	-0.935	0.381	0.917
3b (field 8)	Student's *t*-test	7	-0.913	0.392	0.166
3b (field 9)	Student's *t*-test	6	-0.696	0.512	0.667
3b (field 10)	Student's *t*-test	7	0.580	0.580	0.315
**FIGURE 4**					
4 a	Student's *t*-test	11	-0.298	0.771	0.288
4 b	Student's *t*-test	11	-2.349	0.039	0.212
4 c	Student's *t*-test	11	1.424	0.182	0.261
4 d	Mann-Whitney	-	-	1	< 0.050
4 e	Student's *t*-test	11	-1.478	0.167	0.913
4 f	Mann-Whitney	-	-	0.004	< 0.050
4 g	Student's *t*-test	7	-0.593	0.572	0.498
4 h	Mann-Whitney	-	-	0.093	< 0.050
**FIGURE 5**					

5 b	Student's *t*-test	6	5.538	0.001	0.743
5 d	Student's *t*-test	6	-2.867	0.029	0.796
**FIGURE 6**					
6 a (AEA)	Student's *t*-test	5	-1.273	0.259	0.325
6 a (2-AG)	Student's *t*-test	5	7.063	0.0009	0.432
6 c (3 months)	Student's *t*-test	4	1.186	0.301	0.525
6 c (6 months)	Student's *t*-test	4	-1.519	0.203	0.824
6 c (12 months)	Student's *t*-test	5	-1.273	0.259	0.325
6 d (3 months)	Student's *t*-test	4	0.252	0.814	0.298
6 d (6 months)	Student's *t*-test	4	-0.198	0.853	0.580
6 d (12 months)	Student's *t*-test	5	7.063	0.0009	0.432

Supplementary Table 2. Linear regression analysis between CB_2 and ECS receptors/enzymes. The table reports the statistical results obtained from linear regression analysis between CB_2 and major elements of the ECS (CB_1, TRPV1, DAGLα, DAGLβ, MAGL, NAPE-PLD, FAAH) for individual animals in 12-month-old WT and TG mice.

	**CB_2**							
	**WT**				**TG**			
	**f (x)**	**r**	**R^2**	**P value**	**f (x)**	**r**	**R^2**	**P value**
**CB_1**	Y=2,226 – 1,213 x	0,459	0,211	0,436	y=0,192 + 0,539 x	0,717	0,514	0,563
**TRPV1**	y=1,11-0,13 x	0,455	0,207	0,118	y=1,060 – 0,110 x	0,344	0,118	0,405
**DAGL*α***	y=0,369 + 0,647 x	0,701	0,492	0,187	y=0,544 + 0,376 x	0,740	0,548	0,036
**DAGL*β***	y=-1,217 + 2,217 x	0,985	0,970	0,002	y=0,467 + 0,484 x	0,927	0,859	<0,001
**MAGL**	y = 0.593 + 0.414 x	0,711	0,506	0,178	y=1.730 - 0.151 x	–0,161	0.026	0.7304
**NAPE-PLD**	y=-0,062 + 1,102 x	0,260	0,067	0,740	y=1,209 – 0,004 x	0,003	0	0,995
**FAAH**	y=0,293 + 0,707 x	0,606	0,367	0,279	y=0,101 + 0,821 x	0,787	0,619	0,021

Supplementary Table 3. Linear regression analysis between MAGL and ECS receptors/enzymes. The table reports the statistical results obtained from linear regression analysis between MAGL and major elements of the ECS (CB_1, TRPV1, DAGLα, DAGLβ, MAGL, NAPE-PLD, FAAH) for individual animals in 12-month-old WT and TG mice.

	**MAGL**							
	**WT**				**TG**			
	**F(x)**	**r**	**R^2**	**P value**	**F(x)**	**r**	**R^2**	**P value**
**CB_1**	y = 0.9742 + 0,026 x	0,114	0,013	0,830	y = 1,799 -0,241 x	-0,203	0,041	0,699
**TRPV1**	y = 1.127- 0,120 x	–0,126	0,016	0,840	y = 3,188- 1,883 x	-0,671	0,450	0,099
**DAGLα**	y = 0,842 + 0,151 x	0,304	0,093	0,558	y = 2.272 -0,683 x	-0,375	0,141	0,407
**DAGLβ**	y = 0,854 + 0,153 x	0,592	0,350	0,294	y = 1,783 -0.243 x	-0,142	0,020	0,761
**NAPE-PLD**	y = 0,979 + 0,023 x	0,203	0,041	0,797	y = 1,826- 0,321 x	-0,345	0,119	0,655
**FAAH**	y = 0,580 + 0,426 x	0,855	0,731	0,065	y = 1,669 -0,129 x	-0,148	0,022	0,752

Supplementary Table 4. List of proteins and membranes used for the Western blot analysis. Proteins probed on the same membranes have similar GAPDH bands.

**Membrane ID**	**Proteins identified by Western blot**
Membrane 1	CB_1 (Fig. 4a), NAPE-PLD (Fig. 4g)
Membrane 2	CB_1 (Fig. 4a), NAPE-PLD (Fig. 4g)
Membrane 3	CB_2 (Fig. 4b), FAAH (Fig. 4h), DAGL-β (Fig. 4e), TRPV1 (Fig. 4c)
Membrane 4	CB_2 (Fig. 4b), FAAH (Fig. 4h), DAGL-β (Fig. 4e), TRPV1 (Fig. 4c)
Membrane 5	DAGL-α (Fig. 4d), MAGL (Fig. 4f), APP (Suppl. Fig. 9)
Membrane 6	DAGL-α (Fig. 4d), MAGL (Fig. 4f), APP (Suppl. Fig. 9)
Membrane 7	MAGL (Fig. 4f)

Supplementary Figure 1. Whole Western blot images of CB_1. The images show original Western blot bands of CB_1 of 12-month-old WT and TG retinas with respective GAPDH housekeeping. The red boxes highlight the bands used as representative in the main text (Figure 4 a).

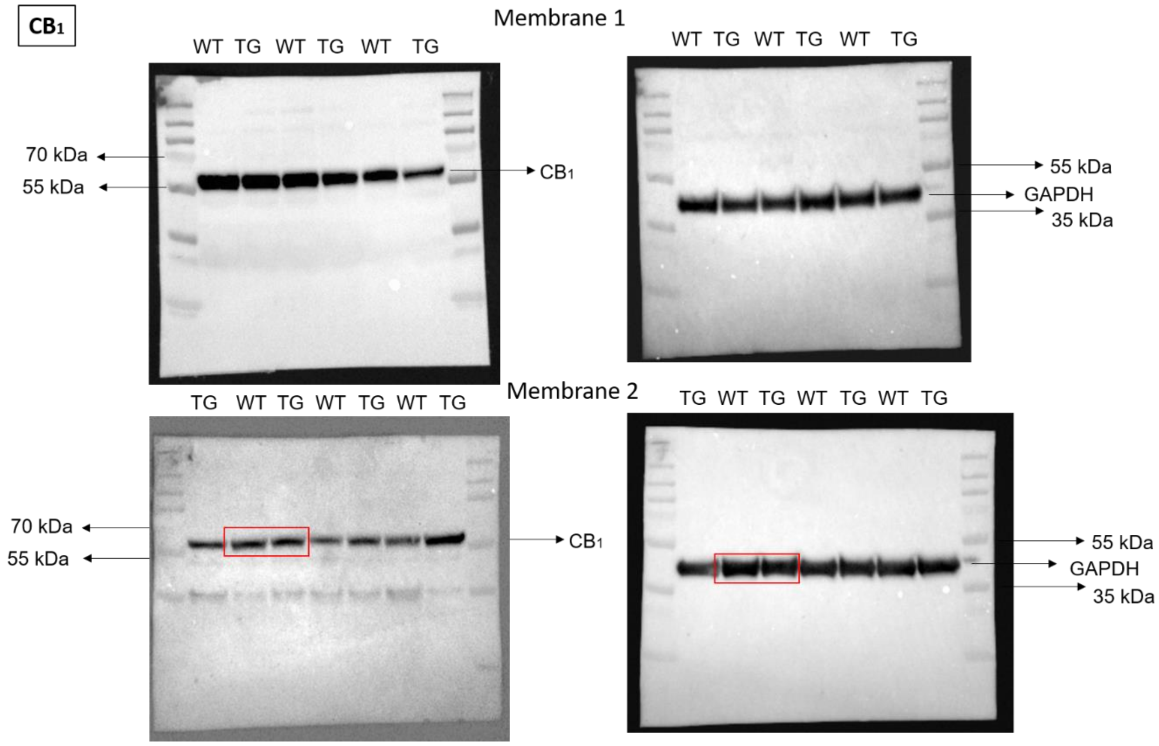

Supplementary Figure 2. Whole Western blot images of CB_2. The images show original Western blot bands of CB_2 of 12-month-old WT and TG retinas with respective GAPDH housekeeping. The red boxes highlight the bands used as representative in the main text (Figure 4 b).

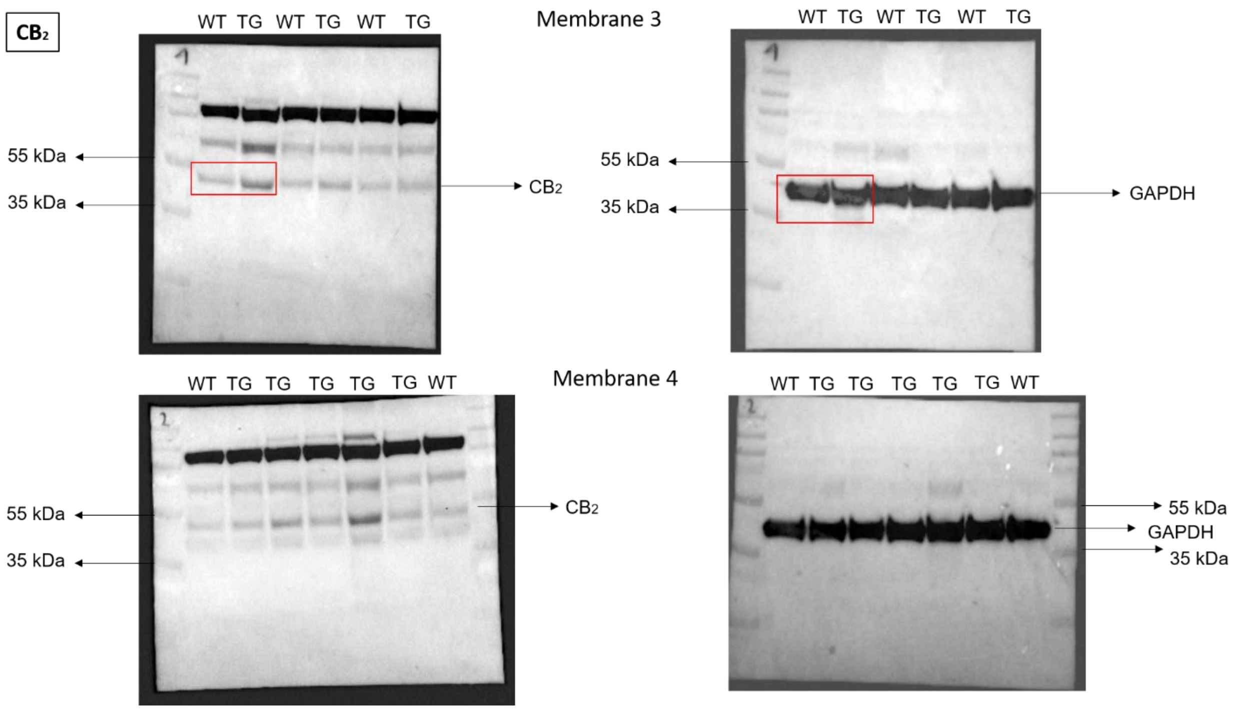

Supplementary Figure 3. Whole Western blot images of TRPV1. The images show original Western blot bands of TRPV1 of 12-month-old WT and TG retinas with respective GAPDH housekeeping. The red boxes highlight the bands used as representative in the main text (Figure 4 c).

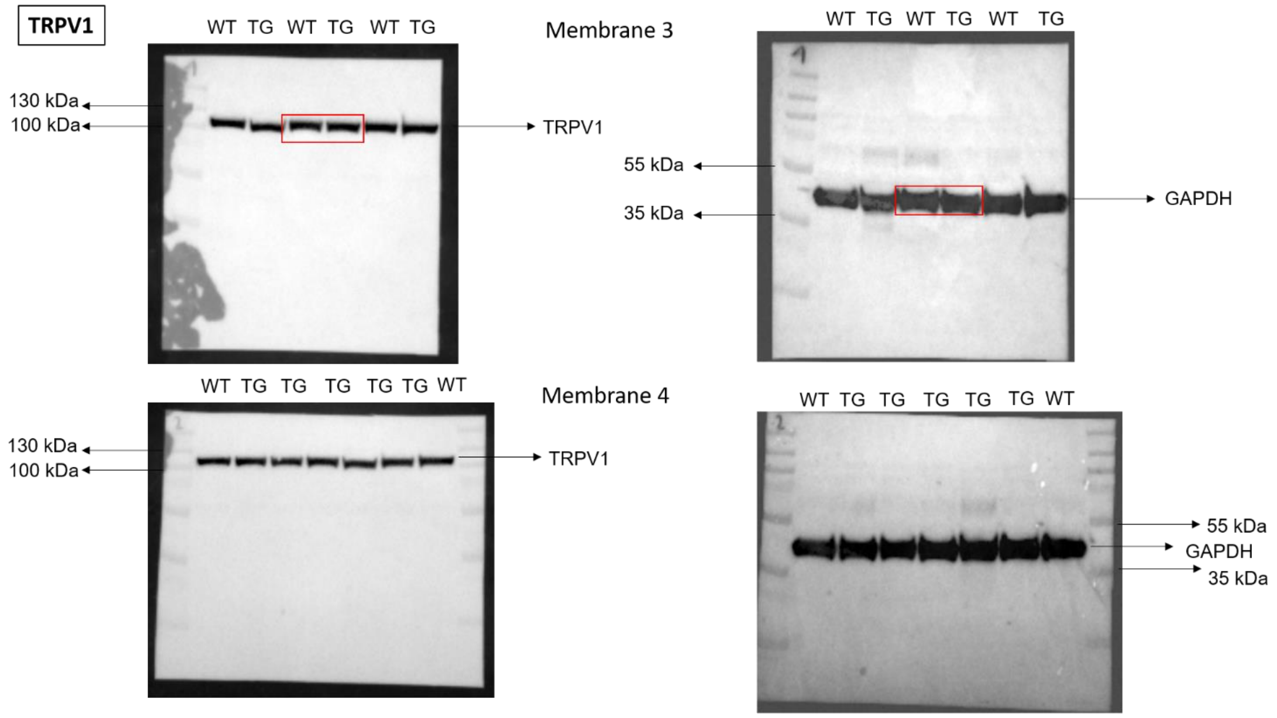

Supplementary Figure 4. Whole Western blot images of DAGL-α. The images show original Western blot bands of DAGL-α of 12-month-old WT and TG retinas with respective GAPDH housekeeping. The red boxes highlight the bands used as representative in the main text (Figure 4 d).

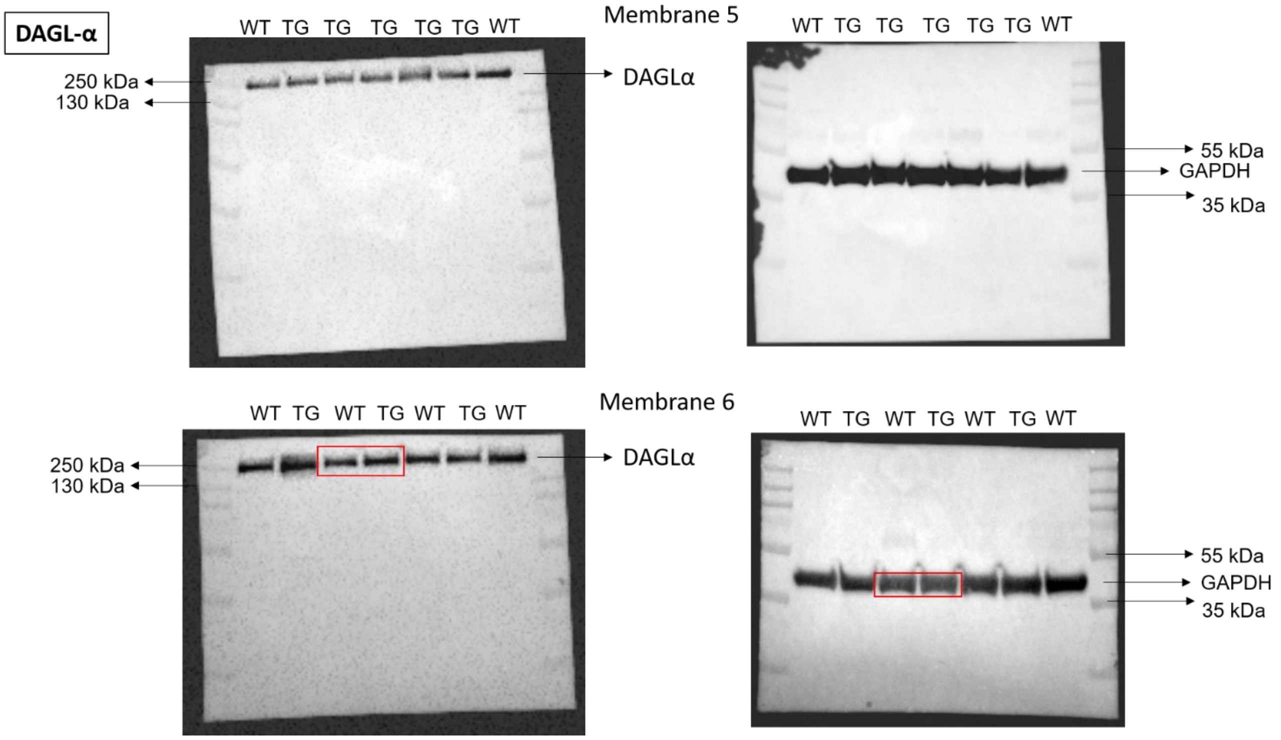

Supplementary Figure 5. Whole Western blot images of DAGL-β. The images show original Western blot bands of DAGL-β of 12-month-old WT and TG retinas with respective GAPDH housekeeping. The red boxes highlight the bands used as representative in the main text (Figure 4 e).

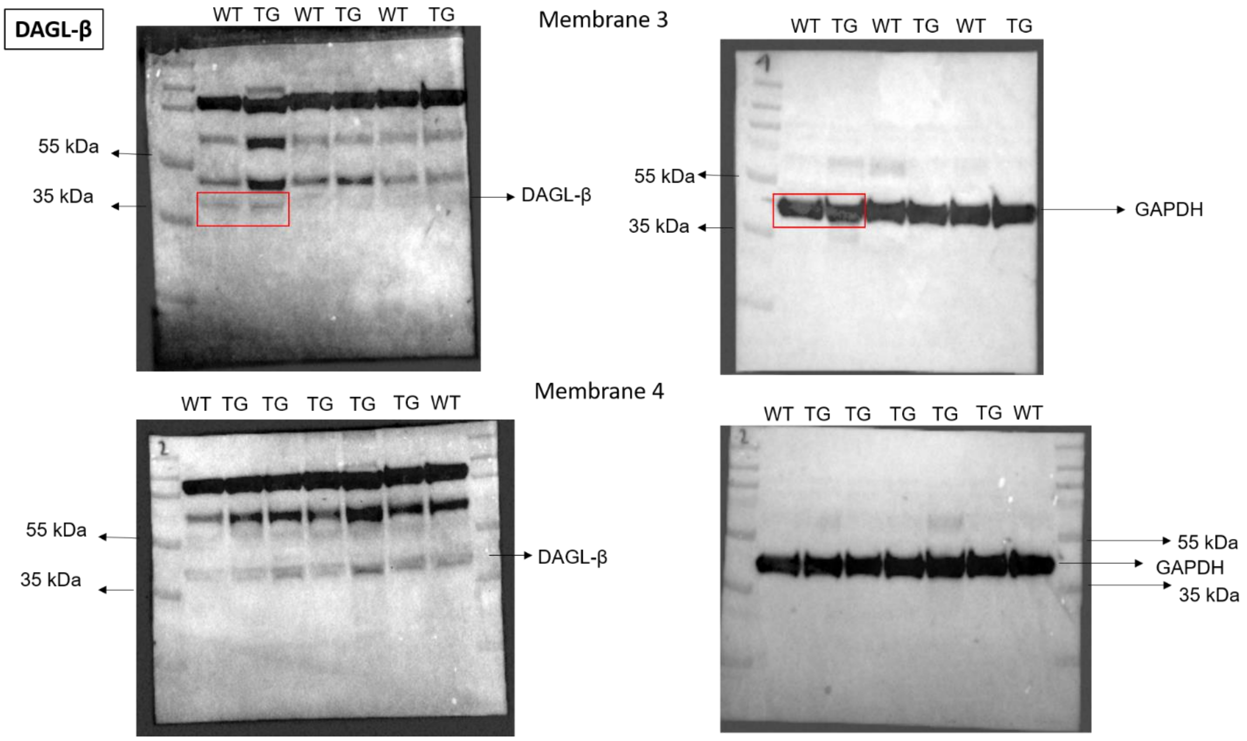

Supplementary Figure 6. Whole Western blot images of MAGL. The images show original Western blot bands of MAGL of 12-month-old WT and TG retinas with respective GAPDH housekeeping. The red boxes highlight the bands used as representative in the main text (Figure 4 f).

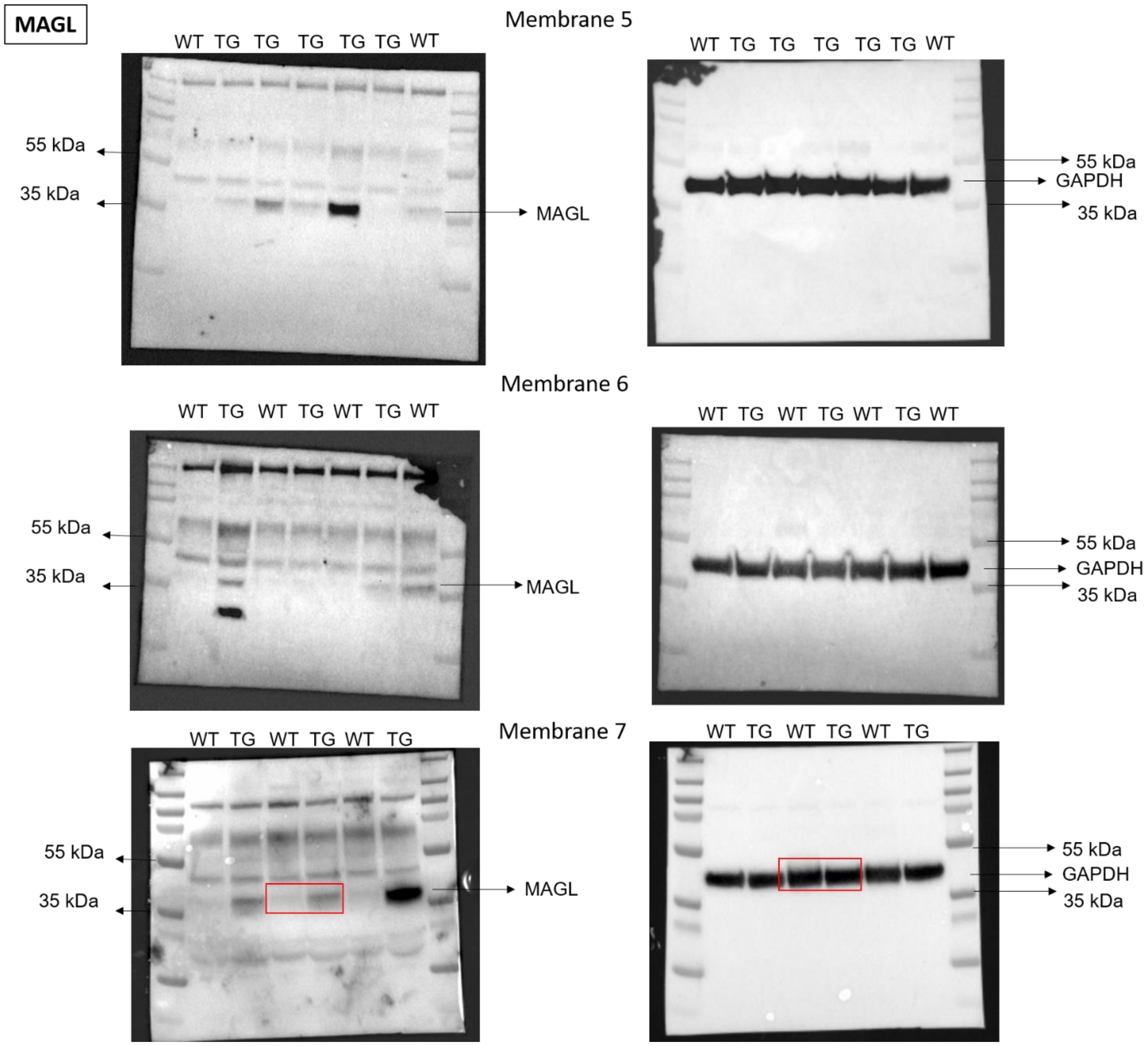

Supplementary Figure 7. Whole Western blot images of NAPE-PLD. The images show original Western blot bands of NAPE-PLD of 12-month-old WT and TG retinas with respective GAPDH housekeeping. The red boxes highlight the bands used as representative in the main text (Figure 4 g).

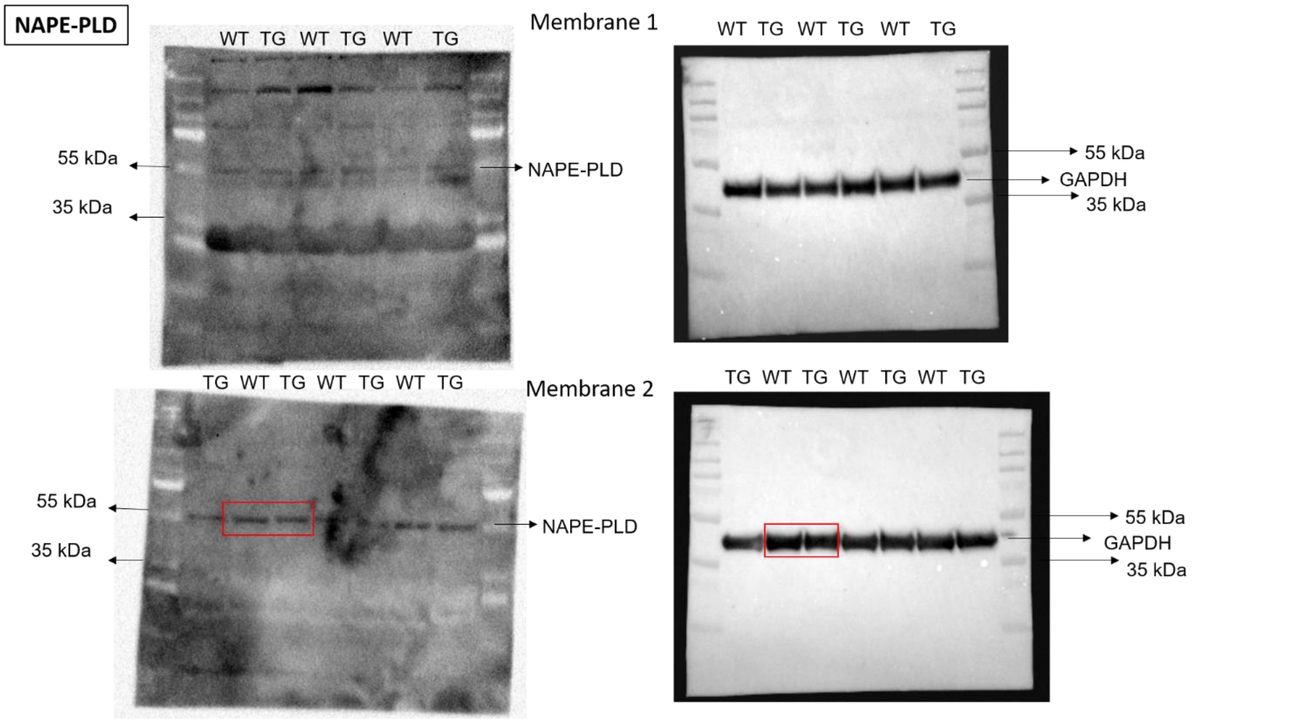

Supplementary Figure 8. Whole Western blot images of FAAH. The images show original Western blot bands of FAAH of 12-month-old WT and TG retinas with respective GAPDH housekeeping. The red boxes highlight the bands used as representative in the main text (Figure 4 h).

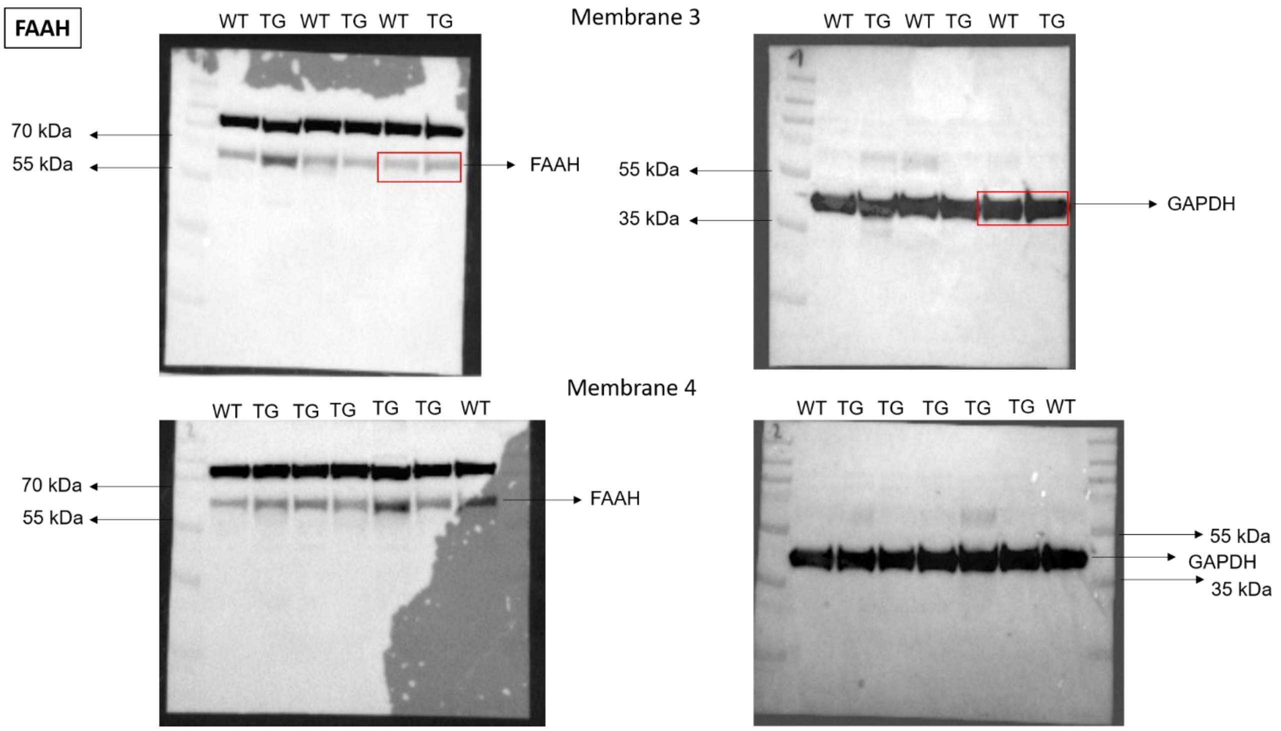

Supplementary Figure 9. Negative control of immunostained cryosections of Figure 5. A retinal cryosections was incubated with 488 anti-rabbit secondary antibody and counterstained with Hoechst (blue).

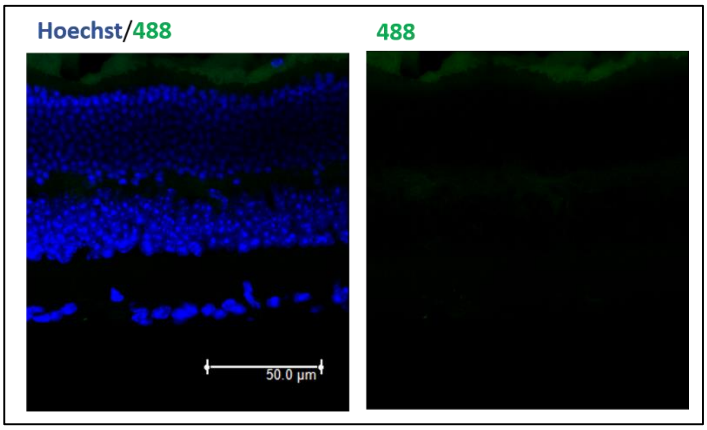

Supplementary Figure 10. Whole Western blot images of APP. The images show original Western blot bands of APP of 12-month-old WT and TG retinas with respective GAPDH housekeeping.

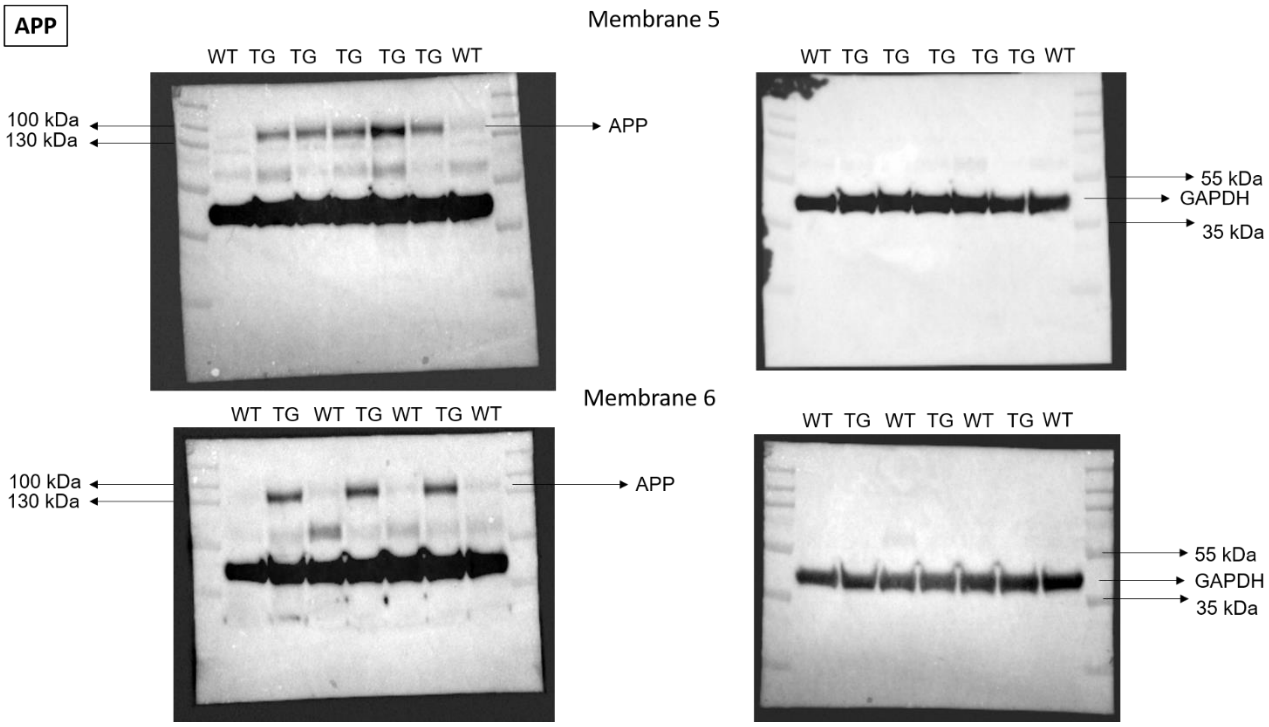

Supplementary Figure 11. Glutamate quantification in the retina of WT and TG mice. Glutamate was quantified in the retinas of 12-month-old WT and TG mice through a bioluminescent Glutamate dehydrogenase/NADH detection system and its content was expressed as μmol/g retina. Data are expressed as mean ± SEM. Data were normally distributed according to Saphiro-Wilk test ($p=0.880$), therefore the Student's *t*-test was used for statistical analysis. No statistically significant differences were found between groups ($t = 0.675$ with 6 degrees of freedom; $p = 0.525$).

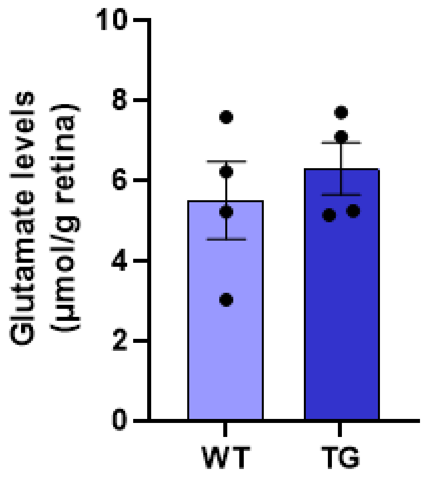

Supplement: Supplementary file 1 — Data S1. [file JNC-169-0-s001.pdf]
